# A scoping review of biopsychosocial risk factors and co-morbidities for common spinal disorders

**DOI:** 10.1371/journal.pone.0197987

**Published:** 2018-06-01

**Authors:** Bart N. Green, Claire D. Johnson, Scott Haldeman, Erin Griffith, Michael B. Clay, Edward J. Kane, Juan M. Castellote, Shanmuganathan Rajasekaran, Matthew Smuck, Eric L. Hurwitz, Kristi Randhawa, Hainan Yu, Margareta Nordin

**Affiliations:** 1 Qualcomm Health Center, Stanford Health Care, San Diego, California, United States of America; 2 Publications Department, National University of Health Sciences, Lombard, Illinois, United States of America; 3 Department of Neurology, University of California, Irvine, California, United States of America; 4 Department of Epidemiology, School of Public Health, University of California, Los Angeles, California, United States of America; 5 World Spine Care, Santa Ana, California, United States of America; 6 Emergency Medicine, Carlsbad, California, United States of America; 7 Rehabilitation Care Line, Physical Medicine and Rehabilitation, Cincinnati Veterans Affairs Medical Center, Cincinnati, Ohio, United States of America; 8 College of Rehabilitative Sciences, Doctor of Physical Therapy Program, University of St. Augustine for Health Sciences, San Marcos, California, United States of America; 9 National School of Occupational Medicine, Carlos III Institute of Health, Complutense University of Madrid, Madrid, Spain; 10 Department of Orthopaedics, Ganga Hospital, Coimbatore, Tamil Nadu, India; 11 Section of Physical Medicine and Rehabilitation and Department of Orthopaedic Surgery, Stanford University, Redwood City, California, United States of America; 12 Office of Public Health Studies, University of Hawai`i, Mānoa, Honolulu, Hawaii, United States of America; 13 UOIT-CMCC Centre for Disability Prevention and Rehabilitation, University of Ontario Institute of Technology, Toronto, Ontario, Canada; 14 Department of Undergraduate Education, Canadian Memorial Chiropractic College, Toronto, Ontario, Canada; 15 Department of Orthopedic Surgery, New York University, New York, New York, United States of America; 16 Department of Environmental Medicine, New York University, New York, New York, United States of America; Stanford University School of Medicine, UNITED STATES

## Abstract

**Objective:**

The purpose of this review was to identify risk factors, prognostic factors, and comorbidities associated with common spinal disorders.

**Methods:**

A scoping review of the literature of common spinal disorders was performed through September 2016. To identify search terms, we developed 3 terminology groups for case definitions: 1) spinal pain of unknown origin, 2) spinal syndromes, and 3) spinal pathology. We used a comprehensive strategy to search PubMed for meta-analyses and systematic reviews of case-control studies, cohort studies, and randomized controlled trials for risk and prognostic factors and cross-sectional studies describing associations and comorbidities.

**Results:**

Of 3,453 candidate papers, 145 met study criteria and were included in this review. Risk factors were reported for group 1: non-specific low back pain (smoking, overweight/obesity, negative recovery expectations), non-specific neck pain (high job demands, monotonous work); group 2: degenerative spinal disease (workers’ compensation claim, degenerative scoliosis), and group 3: spinal tuberculosis (age, imprisonment, previous history of tuberculosis), spinal cord injury (age, accidental injury), vertebral fracture from osteoporosis (type 1 diabetes, certain medications, smoking), and neural tube defects (folic acid deficit, anti-convulsant medications, chlorine, influenza, maternal obesity). A range of comorbidities was identified for spinal disorders.

**Conclusion:**

Many associated factors for common spinal disorders identified in this study are modifiable. The most common spinal disorders are co-morbid with general health conditions, but there is a lack of clarity in the literature differentiating which conditions are merely comorbid versus ones that are risk factors. Modifiable risk factors present opportunities for policy, research, and public health prevention efforts on both the individual patient and community levels. Further research into prevention interventions for spinal disorders is needed to address this gap in the literature.

## Introduction

Spinal disorders include a wide range of musculoskeletal problems affecting the spinal column and associated structures [1]. These disorders are common reasons for patients of all ages and socioeconomic status to seek health care and are a substantial cause of morbidity, disability, and suffering. The prevalence of these disorders has risen sharply since 1990 and they are a leading cause of global years lived with disability [2]. To address these burdens, it would be beneficial to identify risk factors for the most common spinal disorders to inform policy makers and future research efforts of potential public health interventions that might address these risk factors.

Spinal disorders should be considered as components of a more complex biopsychosocial model of health and not in isolation. Population-based studies have suggested that some spinal conditions may be associated with health behaviors, such as smoking [3, 4], high body mass index [5, 6], and insufficient physical activity [7], and with more general health co-morbidities such as anxiety, depression, diabetes, cardiovascular disorders, respiratory problems, and gastrointestinal diseases [8, 9]. Some spinal disorders, such as osteopenia, osteomalacia, and tuberculosis, are affected by factors such as nutrition, living conditions and other psychosocial elements. These disorders are related to comorbidities that are also systemic, such as endocrine disorders and infection. Coexistence of two or more conditions, especially over a long period of time, have been associated with lower quality of life, poorer functional status, and increased utilization of health care [8].

World Spine Care (WSC, www.worldspinecare.org) was established in 2008 with the mission, “to improve lives in underserved communities through sustainable, integrated, evidence-based spinal care.” The WSC vision is to promote, “a world in which everyone has access to the highest quality spine care possible.” The Global Spine Care Initiative (GSCI) is a research proposal created by WSC to reduce the global burden of disease and disability by bringing together leading health care scientists and specialists, government agencies, and other stakeholders to transform the delivery of spine care in underserved and low-income communities worldwide. One of the goals of the GSCI is to provide an evidence-based care pathway and model of care to guide clinicians, policy makers, and public health programs toward a reduction in the burden of spinal disorders. Thus, to understand the general scope of risk factors for commonly presenting spinal disorders, we volunteered to perform a scoping review of this topic so that prevention measures could be considered in a spine care pathway.

While reviews of risk factors, prognostic factors, and comorbidities have been published for individual spinal disorders, we are unaware of any reviews that have cataloged these variables for a range of common spinal disorders. The purpose of this scoping review was to identify risk factors, prognostic factors, and comorbidities associated with the most common spinal disorders that contribute to the greatest burden on society and are likely to be seen by most health care providers globally.

## Methods

### Study design

We performed a scoping review of the literature, according to previously described methods [10–13]. The goal of a scoping review is to provide a broad overview, using pre-identified methods, that is a preliminary assessment of the potential size and expanse of the extant literature [12]. Scoping reviews are particularly useful when investigating topics that are complex and under-studied [12] as they help to discover gaps in the research literature and identify the types of evidence available in the field of study [13]. Unlike systematic reviews, scoping reviews do not focus on a highly specific question [12], do not involve formal qualitative assessment of each paper reviewed [13], and do not provide lengthy critical appraisals of the literature reviewed [10].

Because the research question was intentionally broad, a scoping review was the appropriate methodology. This paper followed Tricco and colleagues’ recommendations of 5 steps for a scoping review: 1) identification of the research question; 2) identification of relevant studies; 3) use of an iterative team approach to study selection and data extraction; 4) charting the data using quantitative and qualitative analysis; 5) summarizing the results to include implications for policy, practice or research [12]. We did not perform stakeholder consultation, which is considered an optional component of scoping reviews and beyond the scope of this study [11, 12]. A review protocol was not included in a registry and we did not attempt to rate the quality of each article or any risk of bias, as this was a scoping review [13, 14].

### Case definition of common spinal disorders

There is no globally recognized single system of taxonomy for spinal disorders published in the peer-reviewed literature. Thus, we operationally defined “spinal disorders” as, “a wide and heterogeneous variety of diseases affecting the vertebrae, intervertebral discs, facet joints, tendons and ligaments, muscles, spinal cord and nerve roots of the spine.”[15] The current paper addressed spinal disorders in any region of the spine and considered the disorders that a wide variety of spine care clinicians often diagnose and treat in clinical practice. Given the wide range of disorders we were asked to review, we felt it necessary to identify major groupings based on diagnoses and used previous publications [15–18] for this purpose.

Thus, the following 3 groups were developed for the purpose of focusing our search strategy:

1. *Spinal pain of unknown origin—Group 1*: Spinal disorders with history and examination findings that do not lead to a specific clinical diagnosis and have no diagnostic imaging, laboratory, or neurodiagnostic findings (eg, lumbalgia). “Spine pain of unknown origin” represents, “… pain for which no other cause has been found or can be attributed.”[18]
2. *Spinal syndromes—Group 2*: Spinal disorders with history and examination findings that lead to a clinical diagnosis (eg, facet syndrome). In this group, diagnostic imaging, laboratory, or neurodiagnostic findings typically do not confirm such disorders.
3. *Spinal pathology—Group 3*: Spinal disorders with history and examination findings that lead to a clinical diagnosis that can be confirmed by diagnostic imaging, laboratory, or neuro-diagnostic findings (eg, osteoarthrosis). Disorders in this category may include substantial pathologies, such as tumors. However, since spinal tumors are uncommon in most clinics and represent the minority of spinal disorders, we did not include tumors or other similarly uncommon disorders [15–17]. Pain disorders that are primarily of psychological origin were not included.

A complete list of disorders included in and excluded from the case definition of common spinal disorders is shown in Table 1.

**Table 1:** Disorders considered in case definition of common spinal disorders; inclusion and exclusion list.

**Spinal pain of unknown origin: Group 1**	**Type**	**Included in this Review**	**Not Included in this Review**
Spinal disorder with history and exam findings not leading to a clinical diagnosis. Diagnostic imaging, laboratory, or neurodiagnostic findings are non-contributory.	Spinal pain of unknown origin	• Neck pain • Thoracic back pain • Low back pain • Coccygodynia	• Not applicable
	Psychogenic spinal pain	• Not applicable	• Psychogenic spinal pain
**Spinal syndromes: Group 2**	**Type**	**Included in this Review**	**Not Included in this Review**
Spinal disorder with history and exam findings leading to a likely clinical diagnosis. Diagnostic imaging, laboratory, or neurodiagnostic findings typically do not confirm such disorders. Syndrome usually improves with treatment targeted at primary pain generator.	Spinal Syndromes	• Radicular pain - sciatica - radiculopathy - brachial plexopathy - neuritis • Joint pain syndromes - costotransverse - zygapophysial - sacroiliac - sacrococcygeal - segmental/somatic dysfunction • Myofascial pain • Soft tissue injury - sprains - strains - whiplash • Torticollis	• Not applicable
**Spinal Pathology: Group 3**	**Type**	**Included in this Review**	**Not Included in this Review**
Spinal disorder with history and exam findings leading to a likely clinical diagnosis confirmed by diagnostic imaging, laboratory, or neurodiagnostic findings.	Arthritis	• Osteoarthrosis	• Arthritides (ankylosing spondylitis, juvenile rheumatoid arthritis, psoriatic arthritis, reactive arthritis, rheumatoid arthritis, seronegative spondyloarthropathy)
	Traumatic	• Dislocation • Subluxation • Fractures • Spinal cord disorders • Myelopathy	• Not applicable
	Infectious	• Tuberculosis	• Arachnoiditis • Epineural fibrosis • Other spinal infectious organisms
	Neoplastic	• Not applicable	• Bone tumors (benign, malignant, metastatic) • Intradural and epidural tumors • Meningeal carcinomatosis • Multiple myeloma
	Metabolic	• Osteopenia	• Osteochondrosis (Scheurmann disease) • Osteitis fibrocystica • Ochronotic spondylosis • Paget disease
	Congenital or developmental	• Scoliosis • Spina bifida • Spondylolisthesis • Vertebral osteochondrosis	• Interspinous pseudoarthrosis • Vertebral epiphysitis
	Referred spinal pain	• Not applicable	• Sickle cell anemia • Lymphoma • Abdominal abscess • Bacterial endocarditis • Carcinomatous lymphadenopathy • Lymphosarcoma • Hodgkin disease • Aortic aneurysm • Embolism of renal artery • Myocardial ischemia • Myocardial infarction • Visceral referred pain

For clarification, disorders in group 2 often improve when treatment is targeted at what is believed to be the primary pain generator. The vast majority of patients (90% or more) seeking primary care for spinal disorders do so for spinal pain syndromes [15–17]. Yet, evidence from various fields shows that while these syndromes may not have specific imaging or laboratory findings they do have clinical findings and responses to treatment that are somewhat reproducible [19]. It has been argued that spinal pain of unknown origin and the various spinal pain syndromes may be the same. However, clinicians attempt to be specific in their diagnoses, use International Classification of Diseases codes that include these terms, and efforts are ongoing to discover if better identification of subsets of spinal pain may have different responses to different interventions [20]. Thus, for the sake of identifying pertinent search terms, we elected to use 2 distinct groups for spinal pain; those with clinical findings and those without clinical findings.

### Search strategy

A computerized search strategy was developed in consultation with a health sciences librarian and reviewed by a second librarian using the Peer Review of Electronic Search Strategies checklist [21]. Search terms consisted of subject headings specific to each database (eg, MeSH for MEDLINE), free text words relevant to this review, and terms based on the research purpose statement, case definition of common spinal disorders, and drawing liberally from the taxonomy by Haldeman et al [17]. PubMed was searched from the inception through September 9, 2016. The search strategy is presented in S1 Appendix and the reporting format followed the PRISMA statement, as described in S2 Appendix.

### Eligibility criteria

The following types of papers were eligible for consideration: systematic review (with or without meta-analysis) of cohort, case-control, or cross-sectional studies, or randomized controlled trials; studied humans; published in the English language; and in a peer-reviewed journal indexed in PubMed. All world regions and all countries and levels of income were included. If no systematic reviews were located for a type of spinal disorder in a term category, then we expanded the search to include case-control or cohort studies for the most recent 10 years (2006–2016) that included risk or prognostic factors calculated with measures of association. To be included in the review, papers were inclusive of adults or children, any demographic, and presented risk or prognostic factors related to morbidity and/or mortality associated with the spinal disorders in the case definition. Cohort, case-control, or randomized controlled trials were used to identify risk or prognostic factors.

The following types of papers were excluded from the review: 1) letters, editorials, commentaries, unpublished manuscripts, dissertations, government reports, books and book chapters, conference proceedings, meeting abstracts, lectures and addresses, consensus development statements; 2) case reports; 3) case series; 4) qualitative studies; 5) non-systematic reviews; 6) papers on post-surgical conditions of the spine; 7) guideline statements; 8) protocols for proposed systematic reviews or meta-analyses; 9) animal studies; and 10) papers that ascertained the effects of drug treatments on outcomes for spinal disorders (dietary studies and supplements were not considered drug treatment).

### Study selection

After the search was conducted, the citations were exported into the EndNote X7 (Thomson Reuters, New York) reference management software program. In the first phase, the lead authors (BNG, CDJ) screened the titles and abstracts for possible relevance, based on the inclusion and exclusion criteria. Possibly relevant papers from the first phase were reviewed in the second phase using the full text article. Articles identified in the references and texts of papers that we read were also considered for inclusion in the present study. The articles were reviewed by the 2 lead authors (BNG, CDJ) and any disagreement was resolved by discussion between the authors to reach consensus. If consensus could not be reached, a third author independently appraised the citation and discussed with the other two authors to reach consensus. Papers not retrieved in the search but suggested by the authoring team were also selected if they met the inclusion criteria.

### Data extraction process

Team members extracted data from the included published papers and recorded the data in a standardized evidence table. Data entry was confirmed by 1 of the 2 lead authors. Risk factors were considered to be any attribute, characteristic, or exposure of an individual that increased the likelihood of developing an incident common spinal disorder or morbidity or mortality as a result of that disorder and were the results of clinical trials, cohort studies, case control studies or reviews of these studies [22, 23]. Prognostic factors were defined as factors that affect or determine the course of a disorder once the person already has the disorder [24]. Associations were gathered from reviews of cross-sectional studies where risk factors could not be identified because outcome variables were not assessed before and after exposure. Comorbidity was defined as any condition that may be associated with the spinal disorder being studied without assigning any condition as an index condition; this approach is often used in primary care and is called “multimorbidity” [25]. Cross-sectional studies were reviewed for potential associations of health behaviors, traits, or comorbidities with spinal disorders. Reviews that mixed the measures of association for cohort, case-control, and cross-sectional studies were not reported as risk or prognostic factors, but as either associations or comorbidities when appropriate.

## Results

### Study selection

**Fig 1:**
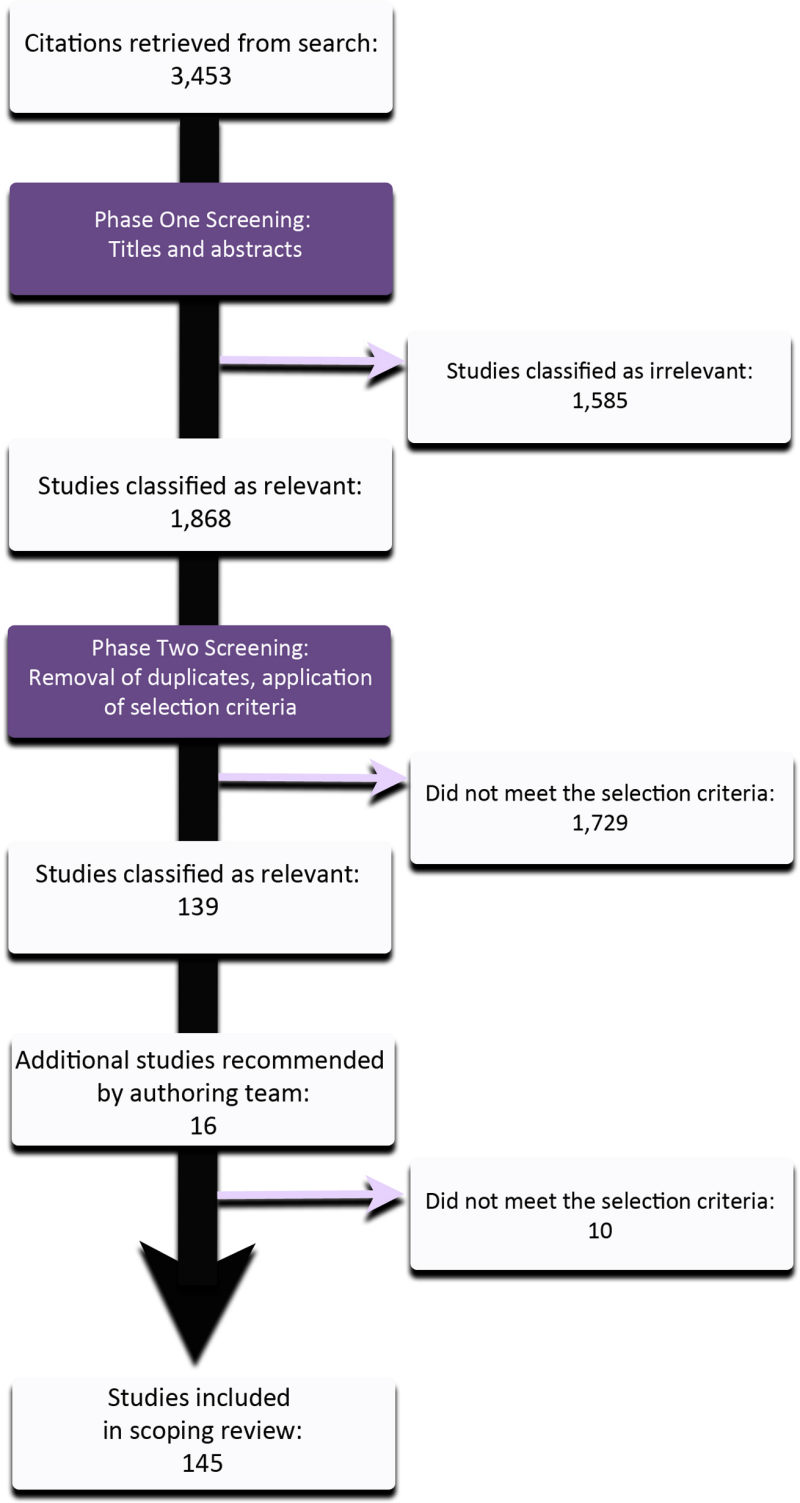
Flow diagram of studies included in this review.

The initial search for systematic reviews and meta-analyses yielded 3,338 studies. There were no systematic reviews for spinal tuberculosis; thus, we searched for primary epidemiological studies published in the past 10 years, which yielded an additional 115 studies, resulting in 3,453 candidate papers. After removing duplicates and citations that were not related to the study question based on review of the titles and abstracts alone, 1,868 studies remained for further scrutiny. Of those, 1,729 were excluded, based on the exclusion criteria, once the full text papers were read. An additional 16 papers were recommended by co-authors, 6 of which met the inclusion criteria. In all, 145 studies were included for final review for scientifically admissible studies (Fig 1).

### Study characteristics and synthesis of results

Systematic reviews were found for each of the term groups of spinal disorders we studied within our term categories, except for spinal tuberculosis, for which we reviewed epidemiological studies.

#### Term group 1—Spinal pain of unknown origin

Sixty-seven studies were reviewed for spinal pain of unknown origin, including 16 for neck pain, 1 for thoracic back pain, and 50 for low back pain. The non-specific neck pain factors were the following (see data in S1 Table):

1. Risk Factors: For neck pain with no specific diagnosis, there was 1 meta-analysis that included only studies with an assessment over time (case-control, cohort) and pooled measures of association [26]. The authors found that the psychosocial work variables of high job demands [pooled OR = 1.17 (95% CI, 1.10–1.24)] and highly monotonous work [pooled OR = 1.30 (95% CI, 1.07–1.57)] were a risk for neck pain.
2. Associations: A meta-analysis was performed only for combat aircrew, for which the generalizability of this study is limited to that population, and included cross-sectional studies, thereby limiting its use for yielding risk factors. However, it was found that jets that are capable of exposing pilots to high gravitational forces had a higher association with neck pain while cumulative flight hours were not associated with neck pain [27]. The remaining papers pertaining to neck pain with no specific diagnosis were 14 systematic reviews [24, 28–39], which did not pool data for meta-analysis, leading authors to provide summaries of each study. The potential associations identified between neck pain and other variables in the systematic reviews were numerous and sometimes conflicted between studies. Commonly mentioned associations with neck pain were: female sex [29–32], psychological status [24, 29, 31, 33, 35], low levels of support at work [31–33, 36], prior history of neck, low back, or other musculoskeletal pain [24, 29–32, 36], prolonged sitting/sedentary work posture [31, 34, 37], poor health [24], no or low levels of physical activity [36, 38], and smoking [29, 31, 32].
3. Comorbidities: Overall ill health [29] and musculoskeletal or general pain in other body sites [39] were mentioned as comorbidities.

The non-specific thoracic back pain factors were the following:

1. Risk factors: There was 1 systematic review pertaining to thoracic back pain of unknown origin and no meta-analyses. The study pertained to children, adolescents, and adults. This review analyzed prospective studies separately from cross-sectional studies and found that for the prospective studies, poorer mental health [OR = 1.4 (95% CI, 1.2–1.9)] and being an older (compared to younger) adolescent [OR = 6.3 (95% CI, 1.2–43.0)] were identified as risk factors for thoracic back pain in adolescence [40].
2. Associations: In children, female sex, postural changes associated with backpack use, and backpack weight were associated with thoracic spine pain. In adolescents, later age of puberty was associated with thoracic back pain. In adults, there was an association between thoracic back pain and difficulty in performing activities of daily living [40].
3. Comorbidities: Comorbidities mentioned for thoracic spine pain included other musculoskeletal symptoms in both adolescents and adults and mental health concerns in adolescents [40].

Ten meta-analyses [3, 26, 27, 41–47] and 39 systematic reviews [28, 38, 39, 48–83] were included pertaining to low back pain with no particular diagnosis. Similar to neck pain, there is a lack of case definition and research method homogeneity among the systematic reviews, leading to inconclusive results pertaining to risk or prognostic factors for back pain. The non-specific low back pain factors were the following (see data in S2 Table):

1. Risk Factors: From the meta-analyses in this review, current [pooled OR = 1.31 (95% CI, 0.11–1.55)] and any history of smoking [pooled OR = 1.32 (95% CI, 0.99–1.77)] were identified as a risk factor for back pain in adults in cohort studies reviewed by Shiri et al [3]. People with acute or subacute pain and negative expectations about their recovery were twice as likely as those with positive expectations to progress to chronic low back pain and have work absenteeism [pooled OR = 2.17 (95% CI, 1.60–2.91)] [44]. Two meta-analyses reported risk factors pertaining to attitudes about back pain and potential psychological risk factors. High job demands [pooled OR = 1.42 (95% CI, 1.19–1.70)], low social support [pooled OR = 1.36 (95% CI, 1.17–1.58)], low supervisor support [pooled OR = 1.33 (95% CI, 1.16–1.53)], and low job satisfaction [pooled OR = 1.31 (95% CI, 1.02–1.69)] [26] were risk factors for back pain, findings that confirmed earlier suspected associations reported in a systematic review by Hoogendoorn et al that linked low job satisfaction with strong evidence of low social support at work and low back pain [62]. Low job control, high job strain, low job security, and highly monotonous work were not [26]. Depressive symptoms were considered a risk factor for low back pain [pooled OR = 1.59 (95% CI, 1.26–2.01)] [47].
2. Associations: Exposure to whole body vibration was associated with low back pain, showing that occupational groups that drive heavy equipment, forklifts, and trucks may be twice as likely to develop low back pain compared to those who do not drive this type of equipment (pooled ORs ranging from 1.39–2.3) [41, 42, 45] Whole body vibration was not associated with abnormal spinal imaging findings in 1 systematic review[49]. Obesity was associated with increased 12-month prevalence of low back pain, seeking care for low back pain, and chronic low back pain in adults [5]. In adult and childhood twins, those in the highest levels of body mass index or weight had 80% higher odds of having low back pain than those in the lower levels [pooled OR = 1.8 (95% CI, 1.6–2.0)] [43]. In a meta-analysis of 40 studies with a representative sample of more than 1 million children, overweight and obesity were associated with an increased risk for low back pain [pooled risk ratio = 1.42 (95% CI, 1.03, 1.97)] [46]. An additional meta-analysis that included cross-sectional studies in its analysis also showed an association between low job satisfaction and low back pain [45]. Other associations with back pain included work-related manual materials handling (measures of association ranging from 1.51–4.1) [45, 58] and frequent bending and twisting (measures of association ranging from 1.6–7.5) [45, 58]. Increased age in workers was associated with low back pain in 1 meta-analysis, showing that those who were age 35–45 yr were 1.5 times as likely to have low back pain than those in the youngest age group and those who were older than 45 yr were nearly twice as likely to have low back pain [45]. A plethora of other associations was reported in the systematic reviews. Many times, conflicting results were reported from the systematic reviews, as was the case with heritability, heavy labor, and various assessments of activities while at leisure.
3. Comorbidities: Comorbidities mentioned for back pain included: psychiatric conditions [53], diabetes [55], headache [55], osteoarthritis [55], osteoporosis [55], chronic fatigue syndrome [55], fibromyalgia [55], cardiovascular conditions [55], sciatica [70], pain at other body sites [39] and other musculoskeletal injuries[46].

#### Term group 2—Spinal syndromes

Eleven papers were included for spinal syndromes, represented by 3 studies on sciatica [41, 84, 85], 6 pertaining to whiplash and its associated disorders [86–91], and 2 on pelvic pain associated with pregnancy [92, 93]. No systematic reviews or meta-analyses were found for any of the other diagnoses or search terms in term group 2. The spinal syndrome factors were the following (see data in S3 Table):

1. Risk Factors: One systematic review found that obesity, overweight, manual labor, and current smoking habit were risk factors for sciatica, although pooled measures of association were not reported due to the study design [84]. The greatest prognostic factor for poor recovery from whiplash associated disorders was a Neck Disability Index score of > 15 [OR = 42.18 (95% CI, 7.37–241.3)] [89]. High initial pain intensity [OR = 5.6 (95% CI, 3.74–8.43)] and catastrophizing [OR = 3.77 (95% CI, 1.33–10.74)] were prognostic factors for poor recovery from whiplash associated disorders [89].
2. Associations: One meta-analysis that included cross-sectional studies in its pooled calculations, showed that whole body vibration experienced in occupational vehicles, such as cranes, fork lifts, and heavy trucks, was associated with sciatica [OR = 2.0 (95% CI, 1.3–2.9)] [41]. A systematic review that included cross-sectional studies in its pooled calculations found that overweight and obesity, smoking, and high levels of physical activity were associated with sciatica [85]. Markers for inflammation (high serum C-reactive protein levels) were potentially associated with sciatica in 1 study [85]. One study of pelvic pain associated with pregnancy found association between pregnancy related pelvic girdle pain and altered motor control and kinematics of the pelvis [92] and another study did not find an association between pregnancy related pelvic girdle pain and serum levels of relaxin hormone [93].
3. Comorbidities: Psychological disorders [86, 91] were mentioned as comorbidities for whiplash associated disorders, as were back pain, headache, widespread chronic pain, degeneration, radicular symptoms, cranial nerve or brainstem disturbance, dizziness, dysphagia, fatigue, and high obesity [90].

#### Term group 3—Spinal pathology

We reviewed the literature on osteoarthritis and associated degenerative spinal conditions. Ten studies were reviewed, including 7 meta-analyses [27, 41, 43, 94–97] and 3 systematic reviews [29, 98, 99]. The osteoarthritis and associated degenerative spinal factors were the following (see data in S4 Table):

1. Risk Factors: Smoking was a risk factor for lumbar disc herniation [relative risk = 1.27 (95% CI, 1.15–1.40)]. Involvement in a workers’ compensation claim was a prognostic factor for poor recovery from neck pain associated with cervical radiculopathy with disc herniation [99]. Scoliotic curve associated with degenerative spine disease was identified as a risk factor in 1 systematic review of prospective studies, although there were no pooled measures of association reported [98].
2. Associations: Males had a higher risk of cervical radicular symptoms if they had cervical disc protrusion when compared to females [29]. In same-sex twins, overweight and obesity were considered risk factors for lumbar disc degeneration; however, this relationship was only true for dizygotic twins [43]. Polymorphisms of vitamin D receptors were reported as potential protective factors in intervertebral disc degeneration [96]. Raastad and colleagues reported that low back pain was associated with different findings on conventional radiographs based on whether samples were drawn from communities or occupations. Disc space narrowing and spondylolisthesis were associated with low back pain in both community and occupation-based samples while spondylolisthesis was significantly more prevalent in the occupation population [pooled OR = 2.21 (95% CI, 1.44–3.39)] [97]. Whole body vibration experienced while driving forklifts, trucks, and other occupational vehicles[41], overweight and obesity [43], were associated with spinal degenerative changes, and tobacco smoking [95]. Disc infection was also associated with degenerative disc disease in 1 study [94].
3. Comorbidities: No comorbidities were identified.

Trauma severe enough to cause fracture and/or dislocation of the spine usually involves traumatic spinal cord injury. The literature on spinal cord injury often reports on both traumatic and non-traumatic etiology of spinal cord injury and we have included data for both etiologies. One meta-analysis and 14 systematic reviews were included [100–114]. Several studies reported prevalence and incidence data for traumatic spinal disorders and identified potential risk factors for injury and prognostic factors for survival; however, few statistical measures of association were reported to support these claims. The spinal cord injury factors were the following (see data in S5 Table):

1. Risk Factors: Reported risk factors for traumatic injury were male sex, age 20–29 yr and ≥ 70 yr [109]. Male sex and advancing age (>75 yr) were risk factors for non-traumatic spinal cord injury [109]. Persons with spinal cord injury have upwards of twice the mortality rate of the general population [112]. Prognostic factors for mortality associated with spinal cord injury were increasing age [pooled HR = 1.06 (95% CI, 1.05–1.07)] and male sex [pooled HR = 1.29 (95% CI, 1.21–1.36)].
2. Associations: Motor vehicle accidents, falls, violence, and sports injuries were reported as being the most common causes of traumatic spinal disorders [103, 107, 109]. Tumors, degeneration, and vascular problems were associated as the most common cause of non-traumatic spinal lesions [109]. As with the meta-analysis, mortality was associated with traumatic more than non-traumatic spinal cord injury, and increased with higher age at injury, pre-existing comorbidities, and higher injury score [108, 110, 111]. Prognostic associations for worse recovery were higher age at lesion onset, damage at higher neurological levels, and the completeness of damage [108, 112, 114]. As time passed following spinal cord injury, the prevalence of pain increased [113].
3. Comorbidities: Pre-existing comorbidities that may be associated with cervical spine injury and spinal cord injury were depression and depressive symptoms [100, 104, 113], anxiety [100], post-traumatic stress disorder [100], high levels of pain [100], chronic pain [101], cardiovascular diseases [102, 111], ankylosing spondylitis [110], osteoarthritis [110], metabolic disorders [110], pulmonary disease [110, 111], gastrointestinal disease [110], renal pathology [110], neoplasia [110], cerebral pathology [110], and peripheral neuropathy [111]. However, the investigation of comorbidities is a nascent area of research in need of stronger reporting methodologies to allow for the calculation of measures of association.

The most recent 10 years of epidemiological papers pertaining to spinal tuberculosis were reviewed since there were no systematic reviews on this topic. Four case-control [115–118] and 4 cohort studies [119–122] were included in this review. The spinal tuberculosis factors were the following (see data in S6 Table):

1. Risk Factors: Risk factors for spinal tuberculosis were not consistent across studies since they were from different geographic regions and represented different populations. Risk factors included age > 35 yr [OR = 4.7 (95% CI, 2.3–9.7)] [115], history of imprisonment [OR = 2.9 (95% CI, 1.3–6.6)] [115], male sex [OR = 1.9 (95% CI, 1.1–3.4)] [115], history of previous tuberculosis infection [OR = 1.9 (95% CI, 1.5–3.9)] [115] and genetic polymorphisms of monocyte chemotactic protein-1 (ORs ranging from 1.3–3.2) [116–118]. Delay in the diagnosis of spinal tuberculosis was identified as a significant prognostic factor for poor treatment outcomes in 1 study [115]. Clinical findings correlating to worsening spinal tuberculosis included severe vertebral collapse, age < 7 yr at time of diagnosis, involvement at the thoracolumbar level, loss of > 2 vertebral bodies, and presence of at-risk signs on radiography [119]. In another cohort, it was found that children < 10 yr of age at time of diagnosis of lumbar tuberculous lesions had significantly worse (*p* < .01) kyphosis, more vertebral bodies involved and had more vertebral body deformities over the course of time than did adolescents > 17 yr [120]. Specific signs of vertebral instability in the tuberculous spine were predictive of significantly worse spinal deformity at 15 year follow up [122]. A later cohort study also showed that at 15 year follow up, those children with a diagnosis of spinal tuberculosis at < 10 yr of age and treated with medication therapy only and had significantly more severe disease and more morphological changes with growth in both the fusion mass and the adjacent segments [121].
2. Associations: No associations are reported due to the lack of systematic reviews and meta-analyses.
3. Comorbidities: Chronic renal failure, diabetes mellitus, and HIV infection were mentioned as comorbidities [115].

There were 22 meta-analyses [123–144] and 1 systematic review [145] included that pertained to metabolic spinal disorders, mainly focused on vertebral fracture associated with osteoporosis or spinal bone mineral density. The risk factors below are in addition to known risk factors for osteoporosis in general, which are female sex, white persons, and older persons [146]. The 2 primary categories of reviews were related to the potential association of diet or vitamin supplementation with fracture or bone mineral density and risk factors for fracture or bone mineral density. The metabolic disorder factors were the following (see data in S7 Table):

1. Risk Factors: Results from 3 reviews of dietary calcium [139] and calcium supplements [126, 139, 144] showed no association with reducing fractures or increasing bone mineral density. Recent meta-analyses showed no reduction in vertebral fracture or improvement in vertebral bone mineral density with supplementation with vitamin D [123, 129, 135]. One earlier study showed no reduction in vertebral fractures associated with all types of vitamin D but did note reduction in vertebral fractures in a sub-group of subjects supplementing with alfacalcidol only [133]. Supplementation with Fluoride showed a reduction in vertebral fractures in adults when using a low dose (< 20mg fluoride equivalents) but not with other doses [142]. Vitamin K supplementation was assessed in 1 study where all reductions in fractures were reported in studies of Japanese populations only; supplementation with phytonadione and menaquinone-4 reduced bone loss; supplementation with menaquinone-4 was associated with a reduction in vertebral fractures [127]. Finally, 1 meta-analysis investigated the effect of isoflavone supplements on reducing vertebral fracture compared to hormone replacement therapy and found the isoflavones to be equally effective [125]. Risk factors for fracture or low bone mineral density were related to pre-existing medical conditions, medications, health behaviors, non-modifiable risk factors, genetics, and socioeconomic variables. For pre-existing medical conditions, the association between diabetes and vertebral fracture risk or bone mineral density was reviewed in 3 studies. In the most recent meta-analysis, the risk for fracture was found to be higher in both men and women with type 1 diabetes mellitus [138]. In an earlier meta-analysis, type 1 or type 2 diabetes were not associated with an increase or decrease in vertebral fracture risk; however, body mass index was associated with bone mineral density in the spine for those with Type 2 but not Type 1 diabetes mellitus [141]. One meta-analysis found an increase in fractures in patients with untreated hyperprolactinemia [128]. With regard to medications, some people take vitamin K antagonists as anticoagulant medications. It was found that people treated with these medications (eg, warfarin) did not have an increased risk of new vertebral fractures [140]. Use of selective serotonin reuptake inhibitors and antidepressants was associated with an increased risk of vertebral fractures in older patients [134] as was the use of proton pump inhibitors for the treatment of gastric disorders [132]. With health behaviors, current smoking status was a risk factor for vertebral fracture risk in adults, however, prior smoking status was not [143]. Two systematic reviews assessed the relationship between exercise and vertebral fracture risk in women and found no reduction in fractures associated with exercise [130, 131].
2. Associations: A couple of studies looked at non-modifiable factors associated with anatomy and physiology. One study found that small vertebral body dimensions were positively associated with osteoporotic vertebral fracture [137]. Biver and colleagues found that fat mass may influence bone metabolism and density through the production of adipokines (leptin, adiponectin, resistin, visfatin) and found that adiponectin was negatively associated with bone density (independent of sex and fat mass) [124]. One meta-analysis investigated the potential for bone mineral density and osteoporotic fractures to be heritable and found that 9 of 150 candidate genes were associated with regulation of BMD and that 4 of these genes affected risk for fracture [136]. Finally, 1 systematic review found evidence that education may have a protective effect on lumbar spine bone mineral density [145].
3. Comorbidities: Comorbidities mentioned for vertebral fracture associated with osteoporosis were low bone mineral density [124] and frailty [126].

Eleven meta-analyses were reviewed for congenital and developmental spinal disorders. The congenital and developmental factors were the following (see data in S8 Table):

1. Risk Factors: Eight papers investigated neural tube defects and spina bifida [147–154] and 3 investigated adolescent idiopathic scoliosis [155–157]. Folic acid supplementation was protective against neural tube defects (pooled relative risk ranging from 0.31 to 0.43) [147]. Two anti-epileptic medications were associated with a risk for neural tube defects. Women taking carbamazepine were more than 2 and a half times as likely as women not taking this medication to have babies with spina bifida [151]. Women taking valproic acid in the first-trimester of pregnancy were at significant risk (pooled ORs ranging from 7.6 to 12.7) for having babies with spina bifida and neural tube defects [150, 154]. First-trimester efavirenz (an antiretroviral medication used to treat and prevent HIV and AIDS) was not associated with congenital anomalies [148]. Other prenatal exposures considered to be risks for neural tube defects included influenza [152] and chlorine [149]. Maternal obesity was associated with about twice the risk for spina bifida and all neural tube defects [153].
2. Associations: For scoliosis, the following were associated with progressive severe deformity: high initial Cobb angle (OR = 7.6), thoracic curve (OR = 2.3), osteopenia (OR = 2.6), age < 13 yr at time of diagnosis (OR = 2.7), and pre-menarche at diagnosis (OR = 4.0) [156]. Investigations into genetic variation showed that 1 polymorphism was not associated with a risk for scoliosis [155] while another polymorphism was associated with such a risk [157].
3. Comorbidities: No comorbidities were mentioned for the congenital disorders studied.

## Discussion

This review is a one source compendium of published risk factors, prognostic factors, and comorbidities for a wide variety of spinal disorders that are commonly seen by health care providers. Rather than looking at disorders individually, we feel that there is value in analyzing the results from this broader perspective.

### Gaps in the literature identified in this review

Of 16 studies of factors associated with neck pain with no specific diagnosis, there was only 1 meta-analysis. Most of the remaining 15 papers combined study designs into systematic reviews (eg, cross-sectional with cohort studies) and therefore were unable to provide pooled data and calculations. From these papers there were also many opposing views on factors pertaining to neck pain. Even less data were available for thoracic back pain. Thus, the bulk of the studies lacked the rigor necessary to make conclusions regarding concrete risk factors for non-specific neck or thoracic back pain. This is an area with fertile grounds for future research.

Low back pain was the most well-studied spinal pain syndrome with several meta-analyses and systematic reviews. However, the literature lacks clear case definitions and homogeneity of reporting associations and contained differences in methodologies. These issues present a significant problem in deriving conclusive risk or prognostic factors or comorbidities for non-specific low back pain. Meta-analyses with pooled measures of association are needed to provide clear data on significant associations between non-specific low back pain and other variables.

There is ample room in the literature for high quality studies on the various spinal pain syndromes in group 2 of our case definition. Of the 14 different clinical diagnoses often reported by spine care clinicians and for which they receive payment for care, there were only 3 with any evidence for risk or prognostic factors or comorbid variables. This begs the question whether these recognized pain syndromes are any different than non-specific spinal pain. Alternatively, perhaps these syndromes have not received enough attention in the literature and therefore each should be more clearly defined. Given the frequency of which these spinal pain syndromes are discussed in journal articles and textbooks, we expected to find more about risk factors, at the very least. Much research can be done in this area.

In group 3 (spinal pathology) specific tissue pathology is required to render a diagnosis, unlike groups 1 and 2. It was not surprising that more definitive risk factors, prognostic factors, and comorbidities were reported for these spinal disorders. This notwithstanding, considering the high prevalence of spinal degenerative disease globally, the literature is quite weak in this area, as it pertains to risk factors. Much work can be done on this topic, especially considering the burgeoning population of ageing citizens world-wide.

The reporting of data on spinal cord injury is muddled. Papers commonly blend data on traumatic and non-traumatic etiologies. Providing a clear focus on traumatic or non-traumatic causes or associations represents an outstanding opportunity for future research. Increased attention to stronger research methods would greatly help this literature. A lack of injury registries, poor reporting systems, and vast differences in methodologies demonstrate how much academic work is desperately needed in this area.

The present study revealed that spinal tuberculosis represents nearly uncharted territory with regard to high quality studies that can provide risk factors, prognostic factors, or comorbidities associated with this severe disease. There were no meta-analyses, systematic reviews, clinical trials or case-control studies on this topic, which is surprising since spinal tuberculosis is relatively common in underserved areas. Many gaps exist in this literature, ranging from common associations across regions/countries, the association of age with this problem, and the emerging field of genomics.

The literature on osteoporosis/osteopenia-related vertebral fractures represented the highest number of meta-analyses and top-tier research that we reviewed. However, even in this area, it is still unclear which supplements and dietary requirements are associated with reductions in the risk for fracture. The association of diabetes with vertebral fracture is also an emerging area of investigation. Considering the rising prevalence of diabetes, this represents an important area of research.

Congenital spinal disorders are often dismissed as being irrelevant to typical clinical practice since they are rarely concerning in the adult patient. However, when considering women of childbearing age and pregnant women in particular, the risk factors associated with fetal congenital anomalies are important. The present review identified that risk factors for scoliosis are virtually unknown and that comorbidities associated with all of the studied congenital spinal disorders are unreported.

Our study found that a number of health behaviors and modifiable risk factors, such as smoking and obesity, were associated with several spinal disorders and across age and sex groups. This suggests that some spinal disorders may be an expression of overall health. In this review, the smallest measures of association were related to various spinal pains and the largest measures of association were those pertaining to congenital anomalies of the spine. There is also sufficient evidence to suggest that in many people with a spinal disorder, substantial and often severe co-morbidities exist. The degree to which these influence health is a nascent area of investigation. Given the lack of definitive treatments for spine-related disability, this deserves further attention. Further investigation of these commonalities pose interesting challenges in the area of research methods, yet may yield exciting results that could be associated with improvements in public health related to spinal disorders. One can hope that results from such work could lead to research that tests hypotheses related to the prevention of common spinal disorders through modifiable risk factors.

### Spinal disorders and multi-causation

Spinal disorders involve multi-causation and multi-morbidity because people and the social structures and communities that they live in are complex. Krieger and others have argued convincingly for epidemiologists involved in studying chronic diseases to consider the condition of interest within the construct of not only comorbidities and demographic variables, but within ecosocial processes, community, and psychosocial frameworks [158, 159]. Krieger states, “Social and biologic plausibility matter; neither alone is sufficient for evaluating explanations of distributions of disease, disability, and death”[160]. New models of causation, such as the black box [161], the web of causation [162], the Chinese box [163], and contrastive [159] approaches perhaps need further exploration by those studying the epidemiology of spinal disorders to provide solutions for the health of the public.

Although we did not limit our methods to underserved areas and low- and middle-income communities, the findings may have relevance to such areas. It has been recognized that chronic diseases, including musculoskeletal disorders, are an increasing burden on low- and middle-income countries [164, 165]. High-income countries may be able to assist low- and middle-income countries with gaining better control of spinal disorders. While all of the spinal disorders reported in the present paper could be examples of this trend, we have selected back pain and spinal cord injury to illustrate the burdens for low- and middle-income countries and how high-income countries may assist.

Back pain is more common in developing countries [166]. Risk and prognostic factors for spinal disorders vary between high-income countries and low-income countries, as do the burdens of musculoskeletal diseases, with low-income countries typically experiencing higher levels of risk [1, 167]. Further, sociodemographic variables (eg, education, income) and modifiable risk factors (eg, tobacco use, alcohol, physical activity) may have a different influence on the amount and perceived burden of spine disorders within various cultural contexts [168]. For example, Williams et al studied back pain across 6 culturally different low-income countries and found that prevalence and levels of back pain intensity varied widely and that there were statistically significant findings between countries [168]. Various work activities present in some low and middle-income countries (eg, carrying loads on one’s head) have been suggested as a potential causes of LBP in such regions but not in others [169, 170].

As a second example from our study, spinal cord injury showed increased risk and prevalence and poorer prognosis in low-income countries. In general, those with spinal cord injuries have reduced life expectancy and worse outcomes in low-income countries when compared to high income countries [112, 171]. Most cases of spinal cord injury are due to traffic accidents and falls [103, 106] and fatal automobile injuries are the highest cause of mortality in people ages 5–44 yr of age [172]. Prevalence and incidence of spinal cord disorders are often under reported in low- and middle-income countries due to a lack of infrastructure and registries for capturing epidemiologic data for this disorder [103, 172]. For example, data on traumatic spinal cord injury are only available for 3 (Sierra Leone, South Africa, Zimbabwe) of more than 4 dozen countries in the African region [103]. Resources used for surveillance of spinal cord injuries in high-income countries may be deployable to low- and middle-income countries. Countries that have successfully lowered the mortality and morbidity associated with traffic injuries through the effective innovation and implementation of safety belt laws and enhanced enforcement of them may have intellectual resources that can be shared with countries struggling with this problem [173]. With better surveillance and registries to gather data on these injuries, particularly traffic injuries, resources can be allocated where prevention might have the most impact.

### Strengths and limitations

We believe that this study is the first of its kind to provide a compendium of spinal disorders as they relate to risk factors, associations, and co-morbidities. Strengths of the study include: a multi-disciplinary and international team participated in this process; the focus was from a practical patient and clinician point of view; and the findings can be used to inform a care pathway and model of care for spinal disorders.

Our study is limited to common spinal disorders for which we found literature meeting the eligibility criteria. It is possible that we did not include a clinical diagnosis in our term categories, as was the case with “discogenic pain”. However, we are reasonably confident that the bulk of the literature and the relevant papers were identified through the lengthy list of search terms used, the breadth of the review, and the similarity in synonyms, such as “radiculopathy” and its association with disc pain. Due to the inherent design methodology for scoping reviews and heterogeneity of the studies included, we did not attempt to rate quality or level of evidence for each article. Also noted was the inconsistency between papers. As this was a scoping review, we included all reviews that provided information about risk factors or association. This likely gives a slightly skewed look at potential risk factors since we report in the text only variables that were risk factors, associations, or comorbidities and variables that were found not to be associated with the given disorder were reported only in the tables. We searched a limited number of databases and it is thus possible we omitted papers indexed in other databases, such as PsychInfo or Scopus. Due to the broad nature of the research question and the inherent design of a scoping review, we were unable to pool the data and perform a meta-analysis to provide more definitive conclusions.

Our conclusions are limited by the results reported in the papers that met inclusion criteria. Many studies did not report measures of association for risk factors. We recorded the risk factor or comorbidity and any corresponding measure of association as reported by the authors. The tables allow us to see where further research is needed to determine if suspected risk factors and comorbidities may be real.

Most of the studies pertaining to term groups 1 and 2 were not from low- or middle-income populations where the risk factors and spinal conditions may be quite different, as well as the magnitude and impact of exposure and intervention effects. In the present review, the scope was very broad and not amenable to a more in-depth investigation into socioeconomic variances across geographic regions, although this would be an excellent study for the future.

Co-morbidity, or multi-morbidity, is a difficult construct to adequately define and operationalize. Two or more diseases can be found together by chance, by selection bias, or by association; the frame of reference (clinical vs research) matters when discussing this topic [25]. We did not seek to determine whether the comorbidities were by chance, selection bias, or association but we did write about them from the clinical frame of reference, since that is the function of the GSCI. We chose not to identify the spinal disorder as the index condition and other associated disorders as comorbidities; instead, we elected to view the conditions from the perspective of the patient and the provider, rather than the researcher.

## Conclusion

This review presents a compendium of risk and prognostic factors and comorbidities for common spinal disorders. Spinal disorders are co-morbid with several general health conditions. However, no substantial body of literature provides clarity as to which conditions are merely comorbid versus risk factors. Modifiable risk factors may be fertile grounds for initiating research on individual or community-based public health programs that may help prevent or mitigate the effects of common spinal disorders. Summarily, public health and prevention considerations for spinal disorders should be considered in care pathways and models of care, however the model should be flexible to adapt to new research in this emerging field.

## Supporting information

S1 Appendix Search strategy used for the literature search. (DOCX)

S2 Appendix PRISMA checklist for the reporting of this literature review. (DOC)

S1 Table Risk factors, associations, and comorbidities for neck pain. (DOCX)

S2 Table Risk factors, associations, and comorbidities for low back pain. (DOCX)

S3 Table Risk factors, associations, and comorbidities for spinal syndromes. (DOCX)

S4 Table Risk factors, associations, and comorbidities for arthritic spinal disorders. (DOCX)

S5 Table Risk factors, associations, and comorbidities for traumatic spinal disorders. (DOCX)

S6 Table Risk factors and comorbidities for spinal tuberculosis. (DOCX)

S7 Table Risk factors, associations, and comorbidities for metabolic spinal disorders. (DOCX)

S8 Table Risk factors, associations, and comorbidities for congenital spinal disorders. (DOCX)
