# Supplementary material for: A scoping review of biopsychosocial risk factors and co-morbidities for common spinal disorders
Source: PLoS One. 2018 Jun 1;13(6):e0197987. doi: 10.1371/journal.pone.0197987 (PMC5983449; doi:10.1371/journal.pone.0197987)
Supplement: S1 Appendix — (DOCX) [file pone.0197987.s001.docx]

# Appendix. Search strategy used for the literature search.

1. exp Whiplash Injuries/

2. exp Neck Injuries/

3. exp Neck Pain/

4. Neck Muscles/in [Injuries]

5. exp Cervical Vertebrae/in [Injuries]

6. exp Radiculopathy/

7. exp Brachial Plexus Neuropathies/

8. exp Torticollis/

9. whiplash.ab,ti.

10. "neck injur*".ab,ti.

11. "neck pain*".ab,ti.

12. "cervical pain*".ab,ti.

13. "neck ache*".ab,ti.

14. "neckache*".ab,ti.

15. "cervicalgia*".ab,ti.

16. "cervicodynia*".ab,ti.

17. "radiculopath*".ab,ti.

18. "brachial plexus neuropath*".ab,ti.

19. torticollis.ab,ti.

20. WAD.ab,ti.

21. NAD.ab,ti.

22. or/1-21

23. exp Back/ab [Abnormalities]

24. exp Back Injuries/

25. exp Back Pain/

26. Coccyx/in [Injuries]

27. Dislocations/

28. Intermittent Claudication/

29. Intervertebral Disc Degeneration/

30. Lumbar Vertebrae/in [Injuries]

31. exp Lumbosacral Plexus/

32. Lumbosacral Region/in [Injuries]

33. Myofascial Pain Syndromes/

34. Neural Tube Defects/

35. Osteoarthritis, Spine/

36. Osteomalacia/

37. Piriformis Muscle Syndrome/

38. Polyradiculopathy/

39. Sacroiliac Joint/in [injuries]

40. Sacrococcygeal Region/

41. Sacrum/ab, in [Abnormalities, Injuries]

42. Sciatica/

43. Scoliosis/

44. Spinal Cord Diseases/

45. Spinal Cord Injuries/

46. Spinal Diseases/

47. Spinal Dysraphism/

48. Spinal Stenosis/

49. Spondylarthropathies/

50. Spondylolisthesis/

51. Tuberculosis/

52. Tuberculosis, Spinal/

53. (avulsed lumbar adj3 (disc* or disk*)).ab,ti.

54. (back adj3 (ache* or injur* or pain*)).ab,ti.

55. (backache* adj3 (injur* or pain*)).ab,ti.

56. (back pain or back-pain).ab,ti.

57. cauda equina syndrome.ab,ti.

58. coccydynia.ab,ti.

59. coccygodynia.ab,ti.

60. coccyx.ab,ti.

61. (disc* adj3 (disease* or extruded or extrusion or degenerat* or displace* or disease* or herniat* or prolapse* or sequestered or slipped)).ab,ti.

62. (disk* adj3 (disease* or extruded or extrusion or degenerat* or displace* or disease* or herniat* or prolapse* or sequestered or slipped)).ab,ti.

63. dorsalgia.ab,ti.

64. (facet adj arthrosis).ab,ti.

65. facet joint pain.ab,ti.

66. intervertebral disc disease.ab,ti.

67. "low* back pain".ab,ti.

68. "low*-back-pain*".ab,ti.

69. (lumbar adj3 (pain or facet or nerve root* or osteoarthritis or radicul* or spinal stenosis or spondylo* or zygapophys*)).ab,ti.

70. lumboischialgia.ab,ti.

71. myelopathy.ab,ti.

72. (myofascial adj3 pain).ab,ti.

73. (neurogenic adj3 claudication).ab,ti.

74. nerve root inflammation.ab,ti.

75. osteomalacia.ab,ti.

76. "Piriformis syndrome*".ab,ti.

77. radiculalgia.ab,ti.

78. radiculitis.ab,ti.

79. (sacral adj2 pain*).ab,ti.

80. (sacrococcygeal adj2 pain*).ab,ti.

81. (sacroiliac or sacro-iliac).ab,ti.

82. "sciatic*".ab,ti.

83. scoliosis.ab,ti.

84. (SI adj joint).ab,ti.

85. spina bifida.ab,ti.

86. (spinal adj2 stenos?s).ab,ti.

87. ((spine or spinal) adj4 "dislocat* or instability or neuritis or osteoarthritis or sprain* or strain* or stenosis or subluxation").ab,ti.

88. "spondylarthropath*".ab,ti.

89. spondylolisthesis.ab,ti.

90. spondylosis.ab,ti.

91. "tailbone adj3 pain*".ab,ti.

92. (tuberculosis or tuberculoses).ab,ti.

93. ("vertebr*" and "fracture* or osteomalacia or osteoporosis").ab,ti.

94. "vertebrogenic adj3 pain*".ab,ti.

95. Thoracic Injuries/

96. "thoracic adj3 pain*".ab,ti.

97. "thoracic adj3 injur*".ab,ti.

98. thoracolumbar.ab,ti.

99. or/23-98

100. 22 or 99

101. Accidents/

102. Accidental Falls/

103. Causality/

104. Comorbidity/

105. Epidemiologic Factors/

106. Epidemiology/

107. Health Behavior/

108. exp Health Services Accessibility/

109. "Growth and Development"/

110. Occupational Diseases/co, ep [Complications, Epidemiology]

111. Prevalence/

112. Risk/

113. Risk Assessment/

114. Risk Factors/

115. Risk-Taking/

116. Sedentary Lifestyle/

117. Stress, Psychological/

118. "accident*".ab,ti.

119. "aetiolog*".ab,ti.

120. (causation or causality or cause*).ab,ti.

121. "comorbidit*".ab,ti.

122. "determinant*".ab,ti.

123. "etiolog*".ab,ti.

124. (growth adj3 development).ab,ti.

125. (health adj5 (behavi* or clinic* or facilit* or initiative* or promot* or status)).ab,ti.

126. incidence.ab,ti.

127. (natural adj3 history).ab,ti.

128. (occupation* adj3 (disease* or exposure*)).ab,ti.

129. pathogenesis.ab,ti.

130. "predictor*".ab,ti.

131. (prevalence or prevalent).ab,ti.

132. progression.ab,ti.

133. prognosis.ab,ti.

134. (risk or risks).ab,ti.

135. "sedentary or inactiv*".ab,ti.

136. (stress adj3 "mental or psychol*").ab,ti.

137. or/101-136

138. Meta-Analysis as Topic/

139. Meta-Analysis.pt.

140. exp Review Literature as Topic/

141. (meta adj analy*).ti,kw.

142. metaanaly*.ti,kw.

143. ((collaborative or integrative or methodologic* or narrative or scoping or systematic) adj3 (review* or overview*)).ti,kw.

144. (cochrane or embase or medline or pubmed).ab.

145. (psychlit or psyclit or psychinfo or psycinfo).ab.

146. (cinahl or cinhal).ab.

147. science citation index.ab.

148. (hand-search* or handsearch or manual search*).ab.

149. or/138-148

150. 100 and 137 and 149

151. limit 150 to english language
