# Supplementary material for: A scoping review of biopsychosocial risk factors and co-morbidities for common spinal disorders
Source: PLoS One. 2018 Jun 1;13(6):e0197987. doi: 10.1371/journal.pone.0197987 (PMC5983449; doi:10.1371/journal.pone.0197987)
Supplement: S1 Table — (DOCX) [file pone.0197987.s003.docx]

**Supplemental Table 1. Reported Risk Factors, Associations, and Comorbidities for Neck Pain of Unknown Origin.**

| **Citation, year** | **Risk Factor [Measure of Association]** | **Comorbidities Mentioned** | **Conclusion** |
| --- | --- | --- | --- |
| Lang, 2012[26]  (MA) | High job demands [pOR^a,c^ = 1.17 (95% CI, 1.10-1.24)]; highly monotonous work [pOR = 1.30 (95% CI, 1.07-1.57)]. | NR^b^ | High job demands and highly monotonous work were associated with neck pain while low job control, high job strain, low social support, low supervisor support, low coworker support were not associated with neck pain. |
| Shiri, 2015[27]  (MA) | For fighter pilots, exposure to high G-capable vs low-G capable aircraft [pOR = 3.2 (95% CI, 2.08-4.67)]; for all pilots, cumulative flight hours [pOR = 1.54 (95% CI, 0.99-2.39)]; | NR | High-G capable jets are a risk for neck pain. Cumulative flight hours are not a risk for neck pain. |
| Ariens, 2000[34]  (SR) | Non-work-related factors:  leisure-time exercise [(OR = 0.89 (95% CI, 0.63-1.25)]; baseball [RR^d^ = 1.05 (95% CI, 0.40-2.75)]; golf [RR = 0.59 (95% CI, 0.21-2.61)]; bowling [RR = 1.63 (95% CI, 0.70-3.83)]; swimming [RR = 0.71 (95% CI, 0.31-1.63)]; diving [RR = 0.96 (95% CI, 0.36-2.52)]; jogging [RR = 0.86 (95% CI, 0.41-1.81)]; aerobics [RR = 0.94 (95% CI, 0.39-2.29)]; racket sports [RR = 1.14 (95% CI, 0.50-2.60)]; playing any of these sports [RR = 0.39 (95% CI, 0.12-1.30)]; use of free weights [RR = 1.87 (95% CI, 0.74-4.74)]; weight lifting (RR = 0.75 (95% CI, 0.31-1.78)]; strenuous muscular activity in leisure time [OR = 0.4 (95% CI, 0.2-0.7)]; annual driving distance 5,000-10,000 km [OR = 0.99 (95% CI, 0.45- 1.76)], 10,000-15 000 km [OR = 1.48 (95% CI, 0.75-2.93)], 15,000-30,000 km [OR = 1.74 (95% CI, 1.01-2.99)], 30,000-50,000 km [OR = 2.10 (95% CI, 1.24-3.54)], >50, 000 km [OR = 2.43 (95% CI, 1.36-4.34)]. Work-related factors: keyboard placed too high [OR = 4.4 (95% CI, 1.1-17.6)]; sitting [OR = 0.94-1.33], sitting > 5 hrs/day [OR = 1.49 (95% CI, 0.86-2.61)], one-quarter of worktime sitting [OR = 2.68 (95% CI, 1.31-5.49)], half of worktime sitting [(OR = 1.92 (95% CI, 0.98-3.79)], three-quarters of worktime sitting [OR = 2.18 (95% CI, 1.11-4.29)], all of worktime sitting [OR = 2.80 (95% CI, 1.40-5.59)]; uncomfortable posture [OR = 1.59-2.42)]; work with office machines > 5 hr/day [OR = 1.65 (95% CI, 1.02-2.67)]; time spent on telephone [OR = 1.4 (95% CI, 1.0-1.8)]; monotonous work [OR = 2.25-2.95]; chair discomfort [OR = 3.5 (95% CI, 1.4- 8.9)]; vibration >7.5 m/s2 [OR = 3.8]; posture of cervical spine rotation [OR = 2.4 (95% CI, 1.5-3.8)], cervical spine flexion [OR = 1.7 (95%CI, 1.0- 3.0)], cervical spine extension [OR = 2.3 (95% CI, 1.5-3.7)]; mismatch of desk and chair heights [OR = 3.0, (p = 0.021)]; bending the neck at work [OR = 3.4, p = 0.012]; physical stress at work age 30-64 yr [OR = 1.35 (95% CI, 1.27-1.42)], age >64 yr [OR = 1.21 (95% CI, 1.08-1.34)]; lifting heavy loads [RR = 1.21 (95% CI, 0.92-1.59)] to [RR = 1.74 (95% CI, 1.09- 2.77)]; monotonous movements [RR = 1.33 (95% CI, 1.04-1.69)] to [RR = 1.73 (95% CI 1.22-2.47)]; twisted postures [RR = 1.26 (95% CI, 0.97-1.63)] to [RR = 1.69 (95% CI, 1.09-2.63)] and [OR = 1.8 (95%, CI 1.2-2.7)] to [OR = 1.9 (95% CI, 1.2-3.2)]; deep forward flexed trunk [RR = 1.33 (95% CI, 1.06-1.68)] to [RR = 1.68 (95% CI, 1.20-2.34)]; hands above shoulder level [RR = 1.17 (95% CI, 0.96-1.44)] to [RR = 1.38 (95% CI, 1.03-1.84)] | NR | “There is some evidence for a positive relationship between the duration of (fixed) sedentary posture at work and neck pain, and there is some evidence for a positive relationship between twisting or bending of the trunk at work and neck pain.” Of 8 sets of risk factors the authors reviewed, they found some evidence for a relationship between the following risk factors and neck pain: sitting posture and twisting or bending of the trunk. However they found inconclusive evidence for a relationship between the following and neck pain: neck rotation, arm force, hand arm vibration, workplace design factors, driving, sports and exercise. |
| Ariens, 2001[33]  (SR) | Non-work-related risk factors  Conflict related to family [OR = 1.8 (95% CI, 1.1-3.0)]; Work-related risk factors:  Conflict related to work [OR = 3.1 (95% CI, 2.0-4.8)]; monotonous work [OR = 2.25-2.95]; overall psychosocial score [OR = 1.89 - 2.57]; poor work content [OR = 1.94-2.47]; low social support [OR = 1.38-2.57]; high psychosocial work load [OR = 1.24-1.49]; mental stress at work [OR = 1.20 (95% CI, 1.12-1.28)] to [OR = 1.27 (95% CI, 1.11-1.46)]; number of hours spent under a deadline per week [OR = 1.7 (95% CI, 1.4-3.0)]; work variance [OR = 1.7 (95% CI, 1.2-2.5)]; routine work lacking decision making opportunities [OR = 4.2 (95% CI, 2.1- 8.6)]; lack of productivity standard [OR = 3.5 (95% CI, 1.5-8.3)]; fear of being replaced by computers [OR = 3.0 (95% CI, 1.5-6.1)]; high information processing demands [OR = 3.0 (95% CI, 1.4-6.2)]; job requires a variety of tasks [OR = 2.9 (95% CI, 1.5-5.8)]; increasing work pressure [OR = 2.4 (95% CI, 1.1-5.5)]; job satisfaction [OR = 1.7 (95% CI, 1.1-2.6)]; limited rest break opportunities [OR = 7.4 (95% CI, 3.1-17.4)]; low influence and control over work [RR = 1.27 (95% CI, 1.00-1.62)] to [RR = 1.30 (95% CI, 0.93-1.81)]; poor supervisor climate [RR = 1.23 (95% CI, 0.99-1.53)] to [RR = 1.29 (95% CI, 0.93-1.79)]; low stimulus from work [RR = 1.33 (95% CI, 1.05-1.67)] to [RR = 1.52 (95% CI, 1.10-2.11)]; poor relationships with fellow workers [RR = 1.19 (95% CI, 0.94-1.50)] to [RR = 1.20 (95% CI, 0.87-1.65)] high psychological work load [RR = 1.52 (95% CI, 1.20-1.94)] to [RR = 1.83 (95% CI, 1.28-2.61)] low work commitment [OR = 1.67 (95% CI, 1.10-2.60]; low support from superiors [OR = 2.08 (95% CI, 1.32-3.26]; high work demand [OR = 1.82 (95% CI, 1.14-2.92)]; higher workload variability [OR = 1.2 (95% CI, 1.0-1.4)] to [OR = 1.78 (95% CI, 1.16-2.73)]; low compared to high control over time [OR = 1.44 (95% CI, 1.07-1.93)]; high compared to low perceived competition [OR = 1.44 (95% CI, 1.08 - 1.91)] | NR | “The results showed some evidence for a positive relationship between neck pain and high quantitative job demands, poor social (coworker) support, low job control, low skill discretion, and low job satisfaction. Inconclusive evidence was found for the relationship between neck pain and poor supervisor support, conflicts at work, low job security, high job strain, and limited rest break opportunities.” |
| Brink, 2013[37]  (SR) | Cervicothoracic angle [OR = 0.83 (95% CI, 0.72-0.95)]; trunk angle [OR = 0.87 (95% CI, 0.79-0.95)]; thoracic flexion [OR = 0.85 (95% CI, 0.76-0.96)]; pelvic tilt [OR = 1.08 (95% CI, 1.00-1.17)]; extreme cervical flexion angle [OR = 2.8 (95% CI, 1.1-7.3)]; extreme cervical flexion and thoracic flexion angles [OR = 2.2 (95% CI, 1.1-5.6)]; sitting 60 - 70 hr per week vs 50 - 60 hr per week [OR = 2.07 (95% CI, 2.11-3.84)] | NR | “Five elements of sitting were identified as relating to UQMP. Those were sitting duration; activities while sitting;  dynamism; and postural angles.” |
| Carroll, 2008, pp S75-S82[24]  (SR) | Prognostic Factors for Neck Pain: Factors associated with prevalent pain at follow-up: age 30-44 yr [OR=1.7], 45-59 yr [OR = 3.9], 60-75 yr [OR = 2.0]; comorbid low back pain [OR = 1.6]; previous neck injury [OR = 1.5]; not working [OR = 1.6]; cycling [OR = 2.4].  Factors associated with 5+ reductions in neck disability: better general health associated with decreased neck disability score [OR = 2.6 for 61-75, OR = 2.06 for 76-100]; being able to count on someone for assistance with daily tasks mostly/always [OR = 2.94]; high use of coping strategies using self-assurance [OR = 4.44]. Factors associated with 2+ reductions in average pain: counting on someone for support with problems [OR = 2.26]; coping using self-assurance [OR = 2.37]; not blaming self [OR = 3.04]. Factors associated with a 2+ reduction in most severe pain: counting on someone for support with problems [OR = 2.08]. Pain coping strategies involving getting angry or frustrated with the situation were associated with fewer decreases in most severe pain [OR = 0.31], average pain [OR = 0.37], and in neck disability [OR = 0.57]. Reduction in neck pain intensity at 12 months predicted by: higher initial pain intensity [OR = 1.05]; higher levels of optimism [OR = 2.95]; lower need to be social [OR = 0.18]; younger age [OR = 0.91]; fewer other symptoms [OR = 0.91]. Men had greater improvements in pain and disability [RR = 1.27 for improvement and 1.47 for resolution]. High exercise group had similar initial pain and lower initial disability; no associations between exercise intensity and reductions in pain or disability. | NR | “Poor health and prior pain episodes are associated with a poorer prognosis; however, the effect of these factors was modest. Psychologic factors are important in prognosis for neck pain in the general population. Poor psychologic health, and worrying, becoming angry, or becoming frustrated in response to neck pain, were associated with poorer prognosis. Greater optimism, coping that involves  self-assurance, and having less need to socialize, were all associated with better prognosis. The impact of psychologic factors was of at least moderate strength.” |
| Carroll, 2008, pp S93-S100[36]  (SR) | Prognostic Factors for Neck Pain in Workers: Being a metal worker predicted slower return to work [RR = 2.12 (95% CI, 1.08-4.17]. Predictors for the absence of chronic pain at 5 years in chronic neck/shoulder pain lasting 6 months or longer with functional limitations: men, high job demands [OR = 1.2 (95% CI, 1.0-1.4)]; no musculoskeletal disease in past [OR = 0.4 (95% CI, 0.30-0.60)]; engaging in sporting activities [OR = 1.5 (95% CI, 1.1-2.1); women, age over 50 yr [OR = 0.6 (95% CI, 0.4-0.8)]; high repetitive work [OR = 1.3 (95% CI, 1.0-1.6)]; high job demands [OR = 1.2 (95% CI, 1.0-1.4)]; no musculoskeletal disease in past [OR = 0.60 (95% CI, 0.50-0.80)]. Factors associated with 12 month period prevalence of neck pain at 4 years: little influence on own work situation [OR = 2.54 (95% CI, 1.17-5.50)]; female gender [OR = 1.99 (95% CI, 1.30-3.02)]; shoulder pain in previous year [OR = 2.56 (95% CI, 1.44-4.55)]. In active duty military personnel surgically treated for cervical disc herniation, there was shorter duration of service [RR = 0.85, p = .02], more likely to be associated with enlisted personnel [RR = 2.9, p = .002]. Two disc level surgery and additional surgeries more likely to be referred for disability. Compensation index did not predict outcome. Workers in nursing homes and homes for the elderly: recurrence of neck pain predicted only by neck pain duration > 3 months in the year prior to baseline [OR = -1.72 (95% CI, 1.22-2.42)]; gender [OR = 1.28 (95% CI, 0.65-2.54)]; age [OR = 0.99 (95% CI, 0.88-1.70] to [OR = 1.28 (95% CI, 0.76-2.15)]. Predictor of disappearance of long-lasting symptoms in sewing machine operators: changing employment [OR = 4.50 (95% CI, 1.37-14.83)]. Predictors of greater days of sick leave due to neck or shoulder pain: blue collar work > 3 days [OR = 6.8 (95% CI, 2.1-22.4); prior sick leave of > 3 days [OR = 6.5 (95% CI, 2.1-20.4); interaction of continuous pain and intensity of pain [OR = 5.2 (95% CI, 1.0-28.1). | NR | “Some factors that were prognostic of poorer outcome of neck pain in the working population were prior musculoskeletal pain, prior sick leave, and occupational type (blue-collar vs. white-collar/metal workers vs. welders/enlisted vs. being an officer). For some occupations (specifically, sewing machine operators), changing jobs was strongly associated with more positive outcomes. Having little (self-perceived) influence over one’s own work situation was the only psychosocial factor studied which had clear prognostic value; however, the effect of this factor was modest. Preliminary evidence that workers who engaged in general exercise and sporting activities were more likely to experience improvement in neck pain. Exercise predicted better outcome, which was discrepant with those from general population samples where exercise did not predict recovery. There was little value in using age as a prognostic factor for recovery from neck pain in workers. However, prior neck pain and prior sick leave were prognostic for poor recovery. |
| Cote, 2008[31]  (SR) | Workers without regular or prolonged neck pain in the past 12 months: high quantitative job demands [RR = 2.1 (95% CI, 1.3-3.6)]; low co-worker support [RR = 2.4 (95% CI, 1.1-5.3)]; low decision authority [RR = 1.6 (95% CI, 0.7-3.5). Workers: sitting 1-50% of the time [RR = 1.8 (95% CI, 0.9-3.7)] sitting > 95% of the time [RR = 2.3 (95% CI, 1.1-5.2)]; working with neck in flexion ≥ 20o > 70% of the time [RR = 1.6 (95% CI, 0.7-3.8)]; job insecurity [RR = 1.3 (95% CI, 0.9-1.9)]; conflicting job demands [RR = 1.3 (95% CI, 0.7-2.6)]; low skill discretion [RR = 1.3 (95% CI, 0.6-2.7)]. Workers with a “Localized Musculoskeletal Discomfort” score <4: isometric lifting strength of shoulder/neck muscles; high [HRR^e^ = 1], moderate [HRR = 1.2 (95% CI, 0.9-1.6)], low [HRR = 1.3 (95% CI, 1.0-1.7)]; static endurance of neck flexors; high [HRR = 1], moderate [HRR = 1.2 (95% CI, 0.9-1.4)], low [HRR = 1.2 (95% CI, 1.0-1.5)]. Workers with and without neck pain, working at least 24 hours per week: practicing a sport ≥ 10 months per year vs 0 - 3 months per year [OR = 0.82 (95% CI, 0.67-0.99)].  Union members with no or mild neck pain in the past 12 months or moderate pain in the past 7 days: female [RR = 1.9 (95% CI, 1.1-3.3); working with mouse ≥ 30 hr/wk vs. 0-9 [RR = 2.4 (95% CI, 0.8-6.8); working at keyboard ≥ 15 hr/wk vs. 0-9 [RR = 1.8 (95% CI, 0.8-3.9); high job demands [RR = 1.7 (95% CI, 1.0-2.7); low social support [RR = 1.5 (95% CI, 0.9-2.4); pain due to an accident [RR = 3.4 (95% CI, 1.3-9.5); low job control [RR = 1.3 (95% CI, 0.8-2.2); not satisfied with workplace design [RR = 1.4 (95% CI, 0.7-2.9); negative affectivity [RR = 1.3 (95% CI, 0.7-2.5); type A personality [RR = 1.7 (95% CI, 0.9-3.1). Workers with neck/shoulder pain ≥ 6 months with functional limitations (men): born in 1953 [OR = 1]; born in 1945 [OR = 1.5 (95% CI, 1.2-1.9); born in 1938 [OR = 2.0 (95% CI, 1.6-2.6); repetitive work under time constraints at follow up [OR = 1.3 (95% CI, 1.0-1.7); awkward work at follow up [OR = 1.3 (95% CI, 1.1-1.7); high job demand [OR = 1.2 (95% CI, 1.0-1.4); depressive symptoms [OR = 1.3 (95% CI, 1.0-1.8)]; musculoskeletal disease in the past [OR = 1.5 (95% CI, 1.3-1.8)]; sporting activities [OR = 0.8 (95% CI, 0.7-0.9)]; smoking before follow up [OR = 1.2 (95% CI, 1.0-1.4). Workers with neck/shoulder pain ≥ 6 months with functional limitations (women): born in 1953 [OR = 1]; born in 1948 [OR = 1.2 (95% CI, 1.0-1.5)]; born in 1945 [OR = 1.6 (95% CI, 1.3-2.0)]; born in 1938 [OR = 1.5 (95% CI, 1.2-2.0)]; repetitive work under time constraints at follow up [OR = 1.3 (95% CI, 1.0-1.6) vs before [OR = 1.2 (95% CI, 1.0-1.5)]; high job demand [OR = 1.2 (95% CI, 1.0-1.4)], depressive symptoms [OR = 1.5 (95% CI, 1.2-1.9)]; musculoskeletal disease in the past [OR = 1.7 (95% CI, 1.5-2.0)]. Factors predicting neck pain in workers in the past 7 days: great influence on own work situation [OR = 1], some [OR = 1.7 (95% CI, 0.8-3.3)], little/very little [OR = 2.9 (95% CI, 1.2-6.7)]; headache [OR = 2.1 (95% CI, 1.1-3.9)]; medium emotional symptoms [OR = 1], low [OR = 5.6 (95% CI, 2.0-15.3)], high [OR = 4.7 (95% CI, 1.7-13.0)]; married/cohabiting vs. single [OR = 3.5 (95% CI, 1.3-9.3)]. Factors predicting neck pain in the past 12 months: little influence on work situation [OR = 2.2 (95% CI, 1.1-4.1)]; headache [OR = 2.5 (95% CI, 1.6-3.8)]; negative affect [OR = 1.9 (95% CI, 1.1-3.2)]. Newly hired employees with neck/shoulder symptoms: age < 30 yr [HRR = 1], 30-39 yr [HRR = 1.6 (95% CI, 1.1-2.4)], > 40 yr [HRR = 1.9 (95% CI, 1.2-3.0)]; female [HRR = 1.7 (95% CI, 1.2-2.6)]; non-white [HRR = 0.7 (95% CI, 0.5-1.0)]; previous neck/shoulder pain [HRR = 3.3 (95% CI, 2.1-5.2)]; low demand/high latitude [HRR = 1]; high demand/high latitude [HRR = 0.81 (95% CI, 0.4-1.5)]; low demand/low latitude [HRR = 0.8 (95% CI, 0.4-1.5)]; high demand/low latitude [HRR = 1.7 (95% CI, 0.9-3.0)]; keyboard to elbow height difference >0 cm [HRR = 1.3 (95% CI, 0.8-2.1)]; keyboard inner elbow angle >121o [HRR = 0.2 (95% CI, 0.0-0.6)]; distance from table to “J” key >17 cm [HRR = 0.7 (95% CI, 0.5-1.2)]; mouse shoulder flexion angle ≤ 25o [HRR = 1], 26o-34o, [HRR = 1.3 (95% CI, 0.8-2.2)], 35o-44o [HRR = 1.7 (95% CI, 1.0-3.0)], >44o [HRR = 1.3 (95% CI, 0.7-2.3)]; monitor head tilt angle >3o [HRR = 1.5 (95% CI, 0.9-2.6)]; chair armrest [HRR = 0.8 (95% CI, 0.5-1.1)]; telephone headrest [HRR = 1.7 (95% CI, 1.0-3.1)]. Computer workers: working at computer 0-25% of the time [OR = 1], 50% of the time [OR = 1.5 (95% CI, 0.7-3.1)], 100% of the time [OR = 1.6 (95% CI, 0.8-3.3)];  Female computer workers previous symptoms [OR = 2.8 (95% CI, 1.9-4.1)]; screen above eye level [OR = 1.5 (95% CI, 1.0-2.2)]; low influence at work [OR = 2.2 (95% CI, 1.3-3.7)]; low vs high social support [RR = 1.4 (95% CI, 1.0-2.0)]; technical problems with computer < 1/month [RR = 1], weekly [RR = 1.3 (95% CI, 0.9-1.8)]; repetitive tasks and movements [RR = 1.3 (95% CI, 1.0-1.79)]; duration of employment ≥4 yr [OR = 1], 1-3 yr at present job [OR = 1.6 (95% CI, 1.1-1.9)]. Male computer workers: previous symptoms [OR = 3.8 (95% CI, 2.3-6.5)]; duration of employment > 3 yr [OR=1], < 1 yr at present job [OR = 2.1 (95% CI, 1.1-3.9)]; “less than good” computer skills [OR = 0.4 (95% CI, 0.1-0.9)]. Finnish municipal employees who had neck pain < 8 days in the past year and worked on video display units > 4 hours per week: female [OR = 2.9 (95% CI, 1.3-2.7)]; poor physical work environment [OR = 2.1 (95% CI, 0.9-4.9)]; poor keyboard position [OR = 2.1 (95% CI, 1.0-4.5)]; current/ex-smoker [OR = 1.9 (95% CI, 0.8-4.3)]; males 25-43 yr of age [OR = 1], 44-51 yr [OR = 2.7], 52-61 yr [OR=2.5]; females 25-43 yr of age [OR = 1], 44-51 yr [OR = 0.2], 52-61 yr [OR = 2.8]. Employees from nursing homes; age <30 yr [OR = 1] 30-39 yr [OR = 1.9 (95% CI, 1.0-3.9)], 40-49 yr [OR = 1.7 (95% CI, 0.8-3.4)], 50-65 yr [OR = 2.1 (95% CI, 1.0-4.4)]; BMI > 30 kg/m2 [OR = 1.8 (95% CI, 1.1-3.1)]; work in awkward postures [OR = 1.8 (95% CI, 1.1-2.8)]; poor-fair general health [OR = 1.6 (95% CI, 1.0-2.4)]; manual material handling [OR = 1.6 (95% CI, 1.0-2.4]; prolonged work in same position [OR = 1.6 (95% CI, 1.1-2.5)]; repetitive movement with hands/arms [OR = 1.5 (95% CI, 1.0-2.2)]. Workers free from neck pain. Men: country of origin other than Sweden [OR = 1.6 (95% CI, 1.0-2.6)]; widowed vs. married [OR = 2.9 (95% CI, 1.2-6.3)]; < 9 vs > 12 years of education [OR = 2.3 (95% CI, 1.5-3.6)]; skilled manual vs. high level occupation [OR = 2.0 (95% CI, 1.3-3.3)]; unskilled manual vs. high level occupation [OR = 1.5 (95% CI, 0.9-2.6)]; pain in low back or arms/hands [OR = 3.7 (95% CI, 2.6-5.3)]. Women: 55-59 yr vs. 45-49 yr of age [OR = 0.6 (95% CI, 0.4-1.0)], 60-64 yr vs. 45-49 yr [OR = 0.6 (95% CI, 0.4-1.0)]; country of origin other than Sweden [OR = 1.8 (95% CI, 1.2-2.9)]; divorced vs. married [OR = 1.4 (95% CI, 1.0-1.9)]; < 9 vs. > 12 years of education [OR = 1.4 (95% CI, 1.0-2.3)]; pain in low back or arms/hands [OR = 2.7 (95% CI, 2.0-3.8)]. Transit vehicle operators: high vs. low psychological demand [HRR = 1.32 (95% CI, 0.9-1.6)]; low vs. high decision latitude [HRR = 1.3 (95% CI, 1.0-1.7)]; moderate vs. high supervisor support [HRR = 1.3 (95% CI, 1.0-1.7)]; low vs. high supervisor support [HRR = 1.4 (95% CI, 1.1-1.9)]; medium vs. high co-worker support [HRR = 1.2 (95% CI, 0.9-1.6)]; low vs. high co-worker support [HRR = 1.3 (95% CI, 1.0-1.7)]; job demand-control-support quadrant is job strain [HRR = 1.3 (95% CI, 1.0-1.6), is iso-strain [HRR = 1.3 (95% CI, 1.0-1.8)].  Female nurses from acute hospitals: age <30 yr [HRR = 1], ≥50 yr [HRR = 1.4 (95% CI, 0.9-2.3)]; BMI ≥ 30 kg/m2 [HRR = 1.3 (95% CI, 0.9-1.3)]; no previous neck pain [HRR = 1], previous neck pain > 1 year before baseline [HRR = 1.6 (95% CI, 1.1-2.3)], previous neck pain < 1 year before baseline [HRR = 2.8 (95% CI, 2.0-3.9)]; duration of previous neck pain was never [HRR = 1], < 1 wk [HRR = 1.7 (95% CI, 1.1-2.5)]; 1-4 wk [HRR = 2.3 (95% CI, 1.5-3.3)], > 4 wk [HRR = 2.6 (95% CI, 1.7-4.0)]. Previous low back pain never [HRR = 1], > 1 year before baseline [HRR = 1.8 (95% CI, 1.2-2.7)], < 1 year before baseline [HRR = 1.9 (95% CI, 1.4-2.7)]; duration of previous low back pain never [HRR = 1], 1-4 weeks [HRR = 1.8 (95% CI, 1.2-2.7)], > 4 weeks [HRR = 2.3 (95% CI, 1.6-3.3)]; job dissatisfaction low [HRR = 1], intermediate [HRR = 1.3 (95% CI, 0.9-1.8)]; high [HRR = 1.3 (95% CI, 0.8-1.8)]; assist patients to move lying to sitting or sitting to lying ≥ 5times/shift [HRR = 1.4 (95% CI, 0.9-2.1); assist patients to mobilize 1-4 times/shift [HRR = 1.4 (95% CI, 1.0-1.9)], ≥5 times/shift [HRR = 1.6 (95% CI, 1.1-2.3)]; move patient around ≥ 5 times/shift [HRR = 1.6 (95% CI, 1.1-2.4)]; reposition ≥5 times/shift [HRR = 1.5 (95% CI, 0.9-2.4)]; transfer in/out of bath ≥ 1 times/shift [HRR = 1.4 (95% CI, 1.0-2.0)]; wash/dress while on chair ≥ 5 times/shift [HRR = 1.7 (95% CI, 1.1-2.8)], wash/dress while in bed ≥5 times/shift [HRR = 1.6 (95% CI, 1.0-2.5)]. Finnish male machine operators, carpenters and office workers with no pain to moderate pain: multivariable model – occupations of carpenters vs. office workers [OR = 1.6 (95% CI, 1.0-2.5)], machine operators vs. office workers [OR = 1.8 (95% CI, 1.1-2.8). Univariate analysis - twisting/bending of the trunk very little [OR=1], moderate [OR = 1.6 (95% CI, 0.9-2.7)], very much [OR = 1.8 (95% CI, 1.2-2.7)]; moderate/poor job satisfaction [OR = 1.3 (95% CI, 0.9-2.0)]; duration of employment < 8 yr [OR=1], > 15 yr [OR = 1.4 (95% CI, 0.9-2.3)]; troubled by draft [OR = 1.7 (95% CI, 1.2-2.5)]. Finnish male machine operators, carpenters and office workers with no pain to severe pain: multivariable model – occupations of machine operators vs. office workers [OR = 3.9 (95% CI, 2.3-6.9)], machine operators vs. carpenters [OR = 2.5 (95% CI, 1.4-4.4)]; age 33-41 yr vs. 25-32 yr [OR = 1.7 (95% CI, 0.9-3.2)], 42-49 yr vs. 25-32 yr [OR = 2.9 (95% CI, 1.5-5.7); current vs. non-smoker [OR = 1.8 (95% CI, 1.0-3.2). Univariate analysis - twisting/bending of the trunk very little [OR = 1], moderate [OR = 1.7 (95% CI, 0.9-3.2)], very much [OR = 1.9 (95% CI, 1.2-3.2)]; moderate/poor job satisfaction [OR = 1.7 (95% CI, 1.1-2.6)]; duration of employment < 8 yr [OR = 1], 8-15 yr [OR = 2.1 (95% CI, 1.2-3.8), >15 yr [OR = 2.1 (95% CI, 1.1-4.0)]. Computer users free of neck pain: age 35-44 vs. 18-34 yr [RR = 1.4 (95% CI, 0.9-2.2)], ≥ 45 vs. 18-34 yr [RR = 1.2 (95% CI, 0.8-1.8)]; muscular tension medium vs. low [RR = 1.3 (95% CI, 0.9-1.9)], high vs. low [RR = 1.6 (95% CI, 1.0-2.5)]; job strain medium vs. low [RR = 1.5 (95% CI, 1.0-2.3)], high vs. low [RR = 1.5 (95% CI, 1.0-2.5)]; physical exposure medium vs. low [RR = 1.4 (95% CI, 1.0-2.2)], high vs. low [RR = 1.3 (95% CI, 0.9-2.0)]; low muscular tension/low job strain [RR = 1], high tension/low strain [RR = 3.3 (95% CI, 1-9.5)], low tension/high strain [RR = 2.5 (95% CI, 0.9-6.8)], high tension/high strain [RR = 4.0 (95% CI, 1.6-10.0)]; low muscular tension/low physical exposure [RR = 1], high tension/low physical [RR = 1.7 (95% CI, 0.9-3.3)], low tension/high physical [RR = 0.8 (95% CI, 0.3-2.6)], high tension/high physical [RR = 1.9 (0.9-4.2)]; low job strain/low physical exposure [RR = 1], high strain/low physical [RR = 1.1 (95% CI, 0.5-2.5)], low strain/high physical [RR = 0.5 (95% CI, 0.1-2.4)], high strain/high physical [RR = 2.7 (95% CI, 1.2-5.9). | NR | “Most neck pain in workers is nontraumatic and that its etiology is multifaceted. While the evidence suggests that several occupational factors are important contributors to the development of neck pain, it is also evident that no single risk factor is sufficient to cause neck pain.” Age, previous musculoskeletal pain, quantitative job demands, social support at work, job insecurity, low physical capacity,  poor computer workstation design and work posture, sedentary work position, repetitive work and precision work were associated with the development of an episode of neck pain. Preliminary evidence suggested the following were associated with neck pain: sex, history of headache, emotional problems, smoking, awkward work postures, physical work environment, ethnicity. |
| Hamberg-van Reenen, 2007[28]  (SR) | Dynamic endurance [OR = 0.3 (95% CI, 0.09-0.85)]; isometric strength of neck muscles [NR]; flexion mobility inverse if equal or less mobility in segment C7-T1 vs. T1-T2 [RR = 3.1 (95% CI, 1.2-8.3)] | NR | Inconclusive evidence for a relation between various physical capacity measures of the neck and neck pain. |
| Hogg-Johnson, 2008[29]  (SR) | Age (yr): 20-46 vs >46 [Incident Rate Ratio = 0.60 (95% CI, 0.38-0.93); Sex: Female [IRR^f^ = 1.98 (95% CI, 1.53-2.58)], male [IRR = 2.30 (95% CI, 1.27–3.87)]; # children: 3 [OR = 1.5 (95% CI, 1.0 –2.1)], > 4 [OR = 1.6 (95% CI, 1.1–2.4)]; Not working vs working [RR = 1.9 (95% CI, 1.2–2.9)]; Prior history of neck pain [RR = 1.7 (95% CI, 1.2–2.5)], low back pain [RR = 1.7 (95% CI, 1.3–2.1)]; Poor psychological status [RR = 1.5 (95% CI, 1.0–2.7)]; Smoking: Active smoker [RR = 1.2 (95% CI, 0.9 –1.5], second-hand smoke [OR = 1.32 (95% CI, 1.00–1.74)]; Heritability [48% (95% CI, 29–67)]; In football players, prior history of neck injury [RR = 5.0 (95% CI, 3.1 –8.2]; In hockey players, penalty for checking other player from the rear [IRR = 0.24 (95% CI, 0.05–1.08)]. | Ill health or disability | Neck pain may be associated with: younger adults; females; having 3 or more children; ill health or disability in those not working; prior history of neck or low back pain; poor psychological status; smoking; exposure to environmental tobacco smoke; heritability; prior history of neck injury; changes in game rules for hockey |
| Kraatz, 2013[35]  (SR) | For the workers studied, the weight of evidence was strong for an incremental effect of job demands, job control, social support, and job strain, on the development of neck and/or shoulder disorders, though the risk estimators for this incremental effect were (usually ranged between 1 and 2). | NR | “There is evidence for an association between psychosocial workplace factors (as measured by the commonly used job demand control model) and neck/shoulder disorders.” |
| McLean, 2010[32]  (SR) | High job demands [RR = 2.14 (95% CI, 1.28-3.58) and RR = 2.14 (95% CI, 1.27-3.60)]; female sex [RR = 1.9 (95% CI, 1.1-3.1) and RR = 1.2 (95% CI, 0.9-1.5)]; low social or work support [RR = 2.43 (95% CI, 1.11-5.29) and OR = 1.76 (95% CI, 1.24-2.50)]; former smoker [OR = 2.37 (95% CI, 0.69-8.07) and OR = 1.80 (95% CI, 1.14-2.82)]; history of low back problems [RR = 1.7 (95% CI, 1.3-2.1) and HRs ranging from 1.9 to 2.6]; history of neck problems [RR = 3.6 (95% CI, 2.1-6.0) and HRs ranging 2.6-3.3; older age [OR = 2.00 (95% CI, 1.15-3.48) and OR = 2.0 (95% CI, 1.6-2.6). | NR | “Strong evidence was found to link female gender, older age, high job demands, low social or work support, being an ex-smoker a history of low back disorders and a history of neck disorders to the development of non specific neck pain.” |
| Nielsen, 2012[39]  (SR) | Neck pain heritability ranged from 24-58% and 1 study found it was greater in males (52%) than females (34%) | Pain at other body sites | Neck pain heritability ranged from 24-58% and 1 study found it was greater in males (52%) than females (34%) |
| Paksaichol, 2013[30]  (SR) | Office Workers: Female [OR = 1.21 (95% CI, 1.16-1.27)]; age (10-year increments) [OR = 1.04 (95% CI, 1.02-1.07)]; mouse usage time (IQR) [OR = 1.04 (95% CI, 1.00-1.09); computer working hr/day [OR = 1.2 (95% CI, 1.0-1.41)]; previous history of complaints [OR = 7.2 (95% CI, 3.8-13.6); irregular head and body posture [OR = 1.1 (95% CI, 1.0-1.21); task difficulty [OR = 1.2 (95% CI, 1.0-1.51). For men: previous neck pain 1-7 days [OR = 3.8 (95% CI, 2.3-6.5)]; duration of employment in same job < 1 yr [OR = 2.1 (95% CI, 1.1-3.9)]; less than good computer skill [OR = 0.4 (95% CI, 0.1-0.9)]. For women: previous neck pain 1 - 7 days [OR = 2.8 (95% CI, 1.9-4.1); influence at work (low) [OR = 2.2 (95% CI, 1.3-3.7)]; screen height above eye level [OR = 1.5 (95% CI, 1.0-2.2)]; high muscular tension [IRR = 1.9 (95% CI, 1.25-2.93)]; high job strain IRR = [1.6 (95% CI, 1.03-2.61)]; high demand [OR = 1.7 (95% CI, 1.0-2.7)]; female [OR = 1.9 (95% CI, 1.1-3.3)]; pain started after accident [OR = 3.4 (95% CI, 1.3-9.5)]; poor placement of keyboard (keyboard-the edge of the table < 15 cm) [OR = 2.1 (95% CI, 1.0-4.5)]; female [OR = 2.9 (95% CI, 1.3-6.7)]; female [HR = 3.07 (95% CI, 1.18-7.99)]. | NR | “Strong evidence was found for female gender and previous history of neck complaints to be predictors of the onset of neck pain. Interestingly, for a large number of factors that have been mentioned in the literature as risk factors for neck pain, such as high physical leisure activity, low social support, and high psychosocial stress, we found no predictive value for future neck pain in office workers.” |
| Palmer, 2007[40]  (SR) | In workers, moderate evidence was found for a causal relation for repetition at the shoulder and for neck flexion allied with repetition. Limited evidence was found for hand–wrist repetition, neck flexion with respect to static loading and force in the absence of repetition, and high job demands, low control, low job support and job strain. | NR | “There is some evidence that neck pain with palpation tenderness is causally related to workplace exposures.” |
| Sitthipornvorakul, 2011[38]  (SR) | Physical activity [NR]. | NR | Physical activity in children and workers is not likely to be associated with neck pain but the quality of evidence for this conclusion was limited. |

^a^p = pooled measures of association from meta-analyses are denoted with a small case p (eg, pOR). Otherwise, reported measures of association are not pooled and are reported as results from individual studies reviewed.

^b^OR = odds ratio

^c^NR = not reported

^d^RR = relative risk

^e^HRR = hazard rate ratio

^f^IRR = incidence rate ratio

^g^HR = hazard ratio
