# Supplementary material for: A scoping review of biopsychosocial risk factors and co-morbidities for common spinal disorders
Source: PLoS One. 2018 Jun 1;13(6):e0197987. doi: 10.1371/journal.pone.0197987 (PMC5983449; doi:10.1371/journal.pone.0197987)
Supplement: S2 Table — (DOCX) [file pone.0197987.s004.docx]

**Supplemental Table 2. Reported Risk Factors, Associations, and Comorbidities for Low Back Pain of Unknown Origin.**

| **Citation, year** | **Risk Factor [Measure of Association]** | **Comorbidities Mentioned** | **Conclusion** |
| --- | --- | --- | --- |
| Bovenzi, 1999[42]  (MA) | Whole body vibration from occupational vehicles [pOR^a,b^ = 2.3 (95% CI, 1.8-2.9)] | NR^c^ | “…clear evidence for an increased risk for LBP disorders in occupations with exposure to WBV.” |
| Burstrom, 2015[43]  (MA) | Exposure to whole body vibration [pOR = 2.17 (95% CI, 1.61-2.91)] for back pain; [pOR = 1.92 (95% CI, 1.28-2.67)] for sciatica | NR | Scientific evidence that exposure to WBV increases the risk of LBP and sciatica. Pooled odds ratio shows a doubled risk for both outcomes (including sciatica) |
| Dario, 2015[44]  (MA) | Highest levels of BMI or weight vs lower BMI [pOR = 1.8 (95% CI, 1.6-2.0)] | NR | Increased risk for low back pain in those twins with highest levels of obesity. Genetics and early environment are possible mechanisms underlying the relationship between obesity and low back pain. |
| Hallegraeff, 2012[45]  (MA) | Acute or subacute pain and negative expectations about their recovery had significantly greater odds of being absent from work at a given more than 12 weeks after onset of pain [pOR = 2.17 (95% CI, 1.6-2.91)] | NR | “The odds that adults with acute or subacute non-specific LBP and negative recovery expectations will remain absent from work due to progression to chronic LBP are two times greater than for those with more positive expectations. |
| Lang, 2012[26]  (MA) | High job demands [pOR = 1.42 (95% CI, 1.19-1.70)]; low social support [pOR = 1.36 (95% CI, 1.17-1.58)]; low supervisor support [pOR = 1.33 (95% CI, 1.16-1.53)]; low job satisfaction [pOR = 1.31 (95% CI, 1.02-1.69) | NR | High job demands , low social support, low supervisor support, and low job satisfaction were associated with back pain while low job control, high job strain, low job security, and highly monotonous work were not. |
| Lotters, 2003[46]  (MA) | Age 35-45 yr [pOR = 1.47 (95% CI, 1.19-1.82)]; age > 45 [pOR = 1.78 (95% CI, 1.42-2.22)]; manual materials handling [pOR = 1.51 (95% CI, 1.31-1.74)]; frequent bending/twisting [pOR = 1.68 (95% CI, 1.41-2.01)]; whole body vibration [pOR = 1.39 (95% CI, 1.24-1.55)]; job dissatisfaction [pOR = 1.30 (95% CI, 1.17-1.45)] | NR | The model is not an etiologic model, but an attributive model for the effect of work on having nonspecific low back pain. The model gives an estimate of the work-relatedness for the individual worker and can be used as a possible tool for directing intervention strategies. |
| Paulis, 2014[47]  (MA) | Overweight and obesity [RR = 1.42 (95% CI, 1.03, 1.97) | Musculoskeletal injury | Overweight is associated with LBP in children, but the quality of evidence is poor |
| Pinheiro, 2015[48]  (MA) | Depression symptoms [pOR = 1.59 (95% CI, 1.26-2.01) | NR | Symptoms of depression increase the likelihood of future LBP, and severe depression increases the risk, suggesting a dose/response relationship. Increased age may result in a more significant effect |
| Shiri, 2010[3]  (MA) | Adult smokers one-month prevalence [pOR = 1.3 (95% CI, 1.16-1.45)]; disabling LBP [pOR = 2.14 (95% CI, 1.11-4.13)]. Adolescent vs adult smokers [pOR = 1.82 (95% CI, 1.42-2.33) | Reduced perfusion and malnutrition | A modest association was found between smoking and LBP |
| Shiri, 2010[5]  (MA) | Obesity associated with increased 12-month prevalence of LBP [pOR = 1.33 (95% CI, 1.14-1.54); seeking care for low back pain [pOR = 1.56 (95% CI, 1.46-1.67)]; chronic low back pain [pOR = 1.43 (95% CI, 1.28-1.60). | NR | “Overweight and obesity increase the risk of low back pain. Overweight and obesity have the strongest association with seeking care for low back pain and chronic low back pain.” |
| Shiri, 2015[27]  (MA) | Fighter pilot vs helicopter or transport pilot [pOR = 0.80 (85% CI, 0.47-1.38)]; | NR | Aircraft platform type not a risk for low back pain. |
| Bakker, 2009[49]  (SR) | Heavy physical work [NR]; Increased risk for LBP in women after lifting/moving objects greater than 25 lb.; Carrying very heavy loads increased risk for LBP; No statistically significant association between heavy work and LBP; Increased risk for LBP in women performing regular sport; Increased risk for LBP in subjects that exercised less than once a week.; No statistically significant associations between sports activities and LBP.; Increased risk for LBP in men who walked or stood during daily activity; Increased risk for LBP in woman who walked/stood more than 2 hrs per day; Increased risk for LBP in subjects engage in do-it-yourself home repair projects; Decreased risk for LBP in gardening/yard work; No statistically significant association for LBP in do it yourself home repair, gardening and hobby activities in men; Decreased risk for LBP for women sitting for more than 2 hrs. No statistically significant association for LBP and sitting. Decreased risk for LBP for riding a forklift truck more than 10 hr per week. Increased risk for driving a car 10 – 14 hr and 15 – 19 hrs per week. No statistically significant association for LBP and whole body vibration as a risk factor. Increased risk of developing LBP if subject bent forward or backward often. Increased risk of developing LBP if subject bent forward for more than 2 hr per day. No statistically significant association for LBP and bending/twisting. |  | Strong evidence that leisure sports or exercises, sitting and prolonged standing and walking are NOT associated with LBP. Conflicting evidence for heavy physical work as risk factor for LBP. Conflicting evidence for sports/exercise, standing/walking, leisure activities, sitting, whole body vibration, bending/twisting, and nursing tasks as risk factor for LBP. |
| Bible, 2012[50]  (SR) | No correlation between vibration exposure and radiographic findings; WBV not found to have any measureable detrimental effect on vertebral height, sagittal plane displacement, or disc height in machine operators’ seats, which were dampened. Unspring work seats resulted in significantly decreased lumbar disc height; Tractor driving vs non-tractor driving farmers. Regular work related LBP significantly more common among tractor driving farmers. However no difference found with MRI on lumbar degeneration.; No significant difference between drivers and cotwins in regard to workload, occupational lifting per day, time working in bent position, commute time or history of smoking.; Helicopter pilots found to have significantly higher number of cervical osteoarthritic changes than both controls and other pilot groups. No significant differences found in the lumbar spine between groups. Age found to be most important factor that led to degenerative changes in all groups; No significant correlation found between vibration exposure and nucleus pulposus degeneration or Modic changes at all lumbar levels. | NR | No causality can be shown between WBV and abnormal spinal imaging findings. Conflicting data “in literature, WBV has not been established as a cause for objective spinal pathological changes on a scientific basis. |
| Campbell, 2010[51]  (SR) | Informal social support and occurrence of spinal pain: inconclusive evidence of an effect of emotional support on risk of spinal pain. Inconsistent findings for the effect of instrumental support on spinal pain. No significant association between social network size and risk of spinal pain. No significant effect of instrumental support on recovery status or lowering pain; emotional support increased the recovery time for those with back pain. | NR | Inconclusive evidence of a relationship between social support and risk of occurrence of spinal pain. Evidence on prognosis is inconsistent. Moderate evidence of an effect of satisfaction with the level of informal social support and psychological outcomes. |
| Campbell, 2013[52]  (SR) | Employment support: greater level increased association with low back pain [OR > 2.0]; lower levels of employment increased association with low back pain: weak association [OR/RR 1.01 – 1.49]; moderate association [OR/RR 1.5 – 1.99] | NR | Employment related support has little to no effect on risk of occurrence but a more notable effect on prognosis for those with back pain. The overall effect is weak for these findings. |
| Chen, 2009[53]  (SR) | Sitting at work [OR = 6.2 (95% CI, 2.2-17.3) | NR | Insufficient evidence to demonstrate that sedentary behavior is a risk factor for developing low back pain. |
| Chou, 2010[54]  (SR) | Nonorganic signs [LR^e^ = 3.0 (95% CI, 1.7-4.6)];  High levels of maladaptive pain coping behavior [LR = 2.5 (95% CI, 2.2-2.8)]; High baseline  functional impairment [LR = 2.1 (95% CI, 1.2-2.7)]; Psychiatric comorbidities [LR = 2.2 (95% CI, 1.9-2.3)]; Low general health status [LR = 1.8 (95% CI, 1.1-2.0)] | Psychiatric conditions | Maladaptive pain coping behaviors, nonorganic signs, functional impairment,  general health status, and presence of psychiatric comorbidities were predictive of persistent disabling low back  pain |
| Ferreira, 2013[55]  (SR) | Alcohol consumption [OR = 1.3 (95% CI, 1.1-1.5)] to [OR = 1.7 (95% CI, 1.2-2.3)] | NR | Alcohol dependence may be associated with low back pain |
| Ferreira, 2013[56]  (SR) | Heritability [0-60%]; smoking [OR = 3.0 (95% CI, 2.8–3.3)]; obesity [OR = 1.9 (95% CI, 1.6–2.2)]; alcohol [NR]; self-assessment of poor health [OR = 3.4 (95% CI, 2.6-4.6); socioeconomic status [NR]; low levels of physical activity [NR] | Diabetes, headache, osteoarthritis and osteoporosis, chronic fatigue  syndrome, fibromyalgia, cardiovascular conditions | “Genetics has an important contribution to LBP prevalence,  but it appears to be dependent on the degree of low back pain severity.” |
| Hamberg-van Reenen, 2007[28]  (SR) | Risk for low back pain. Reduced range in trunk flexion [OR = 2.5 (95% CI, 1.4-4.5)]; max isometric strength [OR = 0.11 (95% CI, 0.02-0.58)]; isometric lifting strength per increase of 20 lbs [RR = 0.99(95% CI, 0.76-1.29)]; static endurance of extensors [RR = 1.2 (95% CI, 0.6-2.6)]; max isometric strength [RR = 0.71 (95% CI, 0.4-1.27)]; dynamic endurance [RR = 0.55 (95% CI, 0.29-1.04)]; static endurance of extensors [OR = 1.4 (95% CI, 0.4-4.2)] | NR | Strong evidence that trunk muscle endurance has no association with risk of LBP. No relationship between isometric strength of extensors and flexors, dynamic strength curled trunk sit up, static endurance of extensors or mobility of trunk flexion with risk of LBP. No relationship between isokinetic peak torque and work per repetition at varying velocities and risk of LBP. No association between max isokinetic lifting strength, psychophysical lifting strength and static endurance and risk of LBP. Inconclusive evidence for an association between risk of LBP and trunk muscle strength or mobility.  No relationship of static endurance of extensors and abdominal muscles and LBP. No relationship between relative max isometric torque by body mass for extensors and flexors as well as dynamic endurance test number of situps in 30 sec. No relationship between max isokinetic peak torque with velocity of 60/sec of extensors, flexors, left rotators and right rotators with LBP. |
| Hartvigsen, 2000[57]  (SR) | Sitting while at work [OR ranged from 0.72-2.13, not significant]; sedentary occupation [OR ranged from 0.38-1.73, not significant) | NR | “The extensive recent epidemiological literature does not support the popular opinion that sitting-while-at-work is associated with LBP.” |
| Heitz, 2009[58]  (SR) | Studies on subacute patients reported 117 predictors, 56 were biomedical (35 modifiable) and 61 psychosocial (51 modifiable).  Studies on chronic patients reported 105 predictors, 44 biomedical (27 modifiable) and 61 psychosocial (40 modifiable) | NR | Comparing risk factors for return to work in 2 populations with a different duration of LBP found that the pattern of risk factor does not change markedly with increasing duration of symptoms. There is a higher rate of modifiable psychosocial factors at earlier stages compared to later stages. |
| Heneweer, 2011[59]  (SR) | Heavy workload [RR range 1.61 (95% CI, 1.08-2.39) to 4.1 (2.7-6.4)]; accumulation of loads or frequency of lifts [RR range 1.4 (95% CI, 1.0-2.1) to 3.26 (95% CI, 1.52-6.98)]; positioning of lumbar spine in a flexed and/or rotated position [RR range 1.6 (95% CI, 1.1-2.3) to 7.5 (95% CI, 2.9-19.6)]; occupational tasks such as manual handling of materials, physical exertion in nursing and exposure to vehicle driving [RR range 1.6 (95% CI, 1.14-2.3) to 6.2 (95% CI, 1.7-23.2)]; physical activities in leisure time (cleaning, gardening) [RR = 0.39 (95% CI, 0.2-0.76)] | NR | Heavy workload and frequency of lifting had a moderate to strong association with LBP.  Flexed, rotated and awkward lumbar positions had a strong association with LBP. Leisure time physical activities, sports and exercise had inconsistent results. |
| Hestbaek, 2003[60]  (SR) | Previous history of LBP vs no previous history [NR] | NR | High rate of relapse of LBP. The risk of LBP was consistently about twice as high for those with a history of low back pain. Low back pain is not a self-limiting condition. |
| Hill, 2010[61]  (SR) | Children: Spine asymmetry in females (hump size, mm) [OR = 1.29 (95% CI, 1.01-1.57)] per one SD hump size difference, [OR = 1.22 (95% CI, 1.03-1.42)] adjusted for gender, [OR = 1.19 (95% CI, 1.00-1.39) adjusted for 14 other variables; Lumbar extensor endurance [OR = 0.5 (95% CI, 0.3-0.8)], [OR = 0.5 (95% CI, 0.3-0.8)] adjusted for gender, [OR = 0.5 (95% CI, 0.3-0.9)] adjusted for gender and baseline low back pain, [OR = 0.5 (95% CI, 0.3-0.9)] adjusted for gender, baseline low back pain and follow-up well-being and physical activity; Lumbar flexion mobility: extensor endurance [OR = 2.2 (95% CI, 1.4-3.6)], [OR = 2.2 (95% CI, 1.3-3.6)] adjusted for gender, [OR = 1.9 (95% CI, 1.2-2.8)] adjusted for gender and baseline low back pain, [OR = 1.9 (95% CI, 1.1-3.2)] adjusted for gender, baseline low back pain, follow-up well-being and physical activity; Lumbar extension mobility: extensor endurance [OR = 3.2 (95% CI, 1.3-8.1)], [OR = 3.2 (95% CI, 1.3-8.3)] adjusted for gender, [OR = 2.4 (95% CI, 0.9-6.2)] adjusted for gender and baseline LBP, [OR = 2.5 (95% CI, 0.9-6.8)] adjusted for gender, baseline low back pain, follow-up well-being and physical activity; Lumbar flexion + extension mobility: extensor endurance[OR = 1.7 (95% CI, 1.2-2.4)], [OR = 1.7 (95% CI, 1.2-2.4)] adjusted for gender, [OR = 1.5 (95% CI, 1.1-2.2)] adjusted for gender and baseline low back pain, [OR = 1.5 (95% CI, 1.1-2.2)] adjusted for gender, baseline low back pain, follow-up well-being and physical activity; >18 vs <5 sporting activities/wk [RR = 1.6 (95% CI, 1.1-2.7)] adjusted for age and gender; Part-time job [RR = 1.5 (95% CI, 1.1-2.1)] adjusted for age and gender; 8. Abdominal pain > 7 days/month [RR = 1.8 (95% CI, 1.1-3.0)] adjusted for age and gender; 9. Psychosocial difficulties [RR = 1.6 (95% CI, 1.1-2.3)] adjusted for age and gender | NR | “No risk factor has been validated in independent investigation and there is no certainty that any factor places children at risk of developing LBP.” |
| Hoogendoorn, 1999[62]  (SR) | Manual materials handling [OR range 1.5-3.1]; bending and twisting [OR = 8.1]; whole body vibration [OR = 4.8]; high level of physical activity [OR rang 1.5-9.8]  . | NR | Moderate evidence for association of heavy physical work and patient handling on low back pain. Strong evidence for relationship of bending and twisting, whole body vibration and manual materials handling with risk for low back pain. No evidence of association between standing, walking, sitting and various aspects of physical load during leisure time and low back pain. |
| Hoogendoorn, 2000[63]  (SR) | Low social support at work and low job satisfaction [RR/OR range 1.3-1.9]; low job satisfaction [RR/OR range 1.7-3.0] | NR | “Evidence for an effect of work-related psychosocial factors, but the evidence for the role of specific factors has not been established yet.” |
| Janwantanakul, 2012[64]  (SR) | Previous low back pain [OR = 2.4 (95% CI, 1.7-3.39)]; low postural risk factors and high job strain for females [OR = 2.53 (95% CI, 0.09-5.85)]; high postural risk factors and low job strain for females [OR = 2.51 (95% CI, 1.23-5.09)]; high postural risk factors and high job strain for females [OR = 5.51 (95% CI, 2.33-13.03)]; prior back pain < 4 on visual analog scale [OR = 3.0 (95% CI, 1.5-6.0)]; prior back pain > 4 on visual analog scale [OR = 10.3 (95% CI, 4.1-25.5)] | NR | Strong evidence for a relationship of previous history of LBP in office workers and risk of LBP. No predictive value for age, daily computer use, workstation ergonomics, social support and job demands on risk of LBP. |
| Jeffries, 2007[65]  (SR) | Adolescents: Genetic factors [OR = 2.81]; low back pain > 8 days in 1 yr during adolescence predictive for probability of having low back pain in the previous year [OR = 1.87 (95% CI, 1.52–2.32)] and persistent LBP [OR = 1.43 (95% CI, 1.18–1.73)] | NR | Lifetime prevalence for low back pain = 4.7% – 72%. Some evidence that idiopathic spinal pain is a risk factor for spinal pain in adulthood. “Idiopathic spinal pain is a significant public health issue, with prevalence figures in adolescence approaching those in adults by around 18 years of age.” |
| Kent, 2008[66]  (SR) | Low back pain: Significant ORs for predictive for activity limitation: prior episodes [pOR = 2.98 (95% CI, 1.42–6.23)]; poor coping strategies [pOR = 3.24 (95% CI, 1.46–7.22)]; general health [pOR = 4.56 (95% CI, 2.19–9.49)]; job satisfaction [pOR = 2.65 (95% CI, 1.27–5.50)]; less social activity [pOR = 2.31 (95% CI, 1.12–4.76)]. Significant ORs for predictive for restricted participation (undefined): older age [pOR = 1.56 (95% CI, 1.05–2.30)]; greater pain intensity [pOR = 1.45 (95% CI, 1.10–1.91)]; high Oswestry score [pOR = 2.69 (95% CI, 1.01–7.15)]. | NR | A considerable degree of heterogeneity was found in the included studies method, statistical approach, and conclusions. The authors feel that this prevents meaningful conclusions from being made on prognostic criteria. |
| Leboeuf-Yde, 1999[67]  (SR) | Smoking [OR range 1.45 (95% CI, 1.33-1.58) to 3.12 (95% CI, 2.3-4.3)] | NR | 51% of the studies found a statistically significant association between smoking and low back pain. Large sample studies found a RR <2 which indicates only a weak ssociation. Smoking is a likely risk factor in low back pain, but does not appear to actually be causal. |
| Leboeuf-Yde, 2000 (pp 226-37)[68]  (SR) | Body weight: 23% of 111 low back pain variables were associated with some measure of weight, but this positive association was only present in 32% of studies. | NR | The association of body weight and LBP was found to be weak at best |
| Leboeuf-Yde, 2000 (pp 343-6)[69]  (SR) | Alcohol consumption was not found to be statistically associated with LBP | NR | The authors question the conclusions of the studies reviewed. |
| Linton, 2001[70]  (SR) | Job satisfaction [NR]; monotonous work [NR]; work relations [NR]; perceived demands [NR]; work content [NR]; control [NR]; pace [NR]; self-reported stress [NR]; perceived ability to do work [NR]; belief that work is dangerous [NR]; perceived emotional effort [NR]. | NR | Job satisfaction and related psychological factors may be associated with LBP. The authors further suggest that an understanding of why each factor is important will likely be necessary to advance prevention of LBP |
| Lis, 2007[71]  (SR) | Sitting [OR range 1.0 (95% CI, 0.5-1.9) to 9.0 (95% CI, 4.9-16.4)] for various occupations | NR | Extended periods of sitting by itself was not associated with a high risk of LBP. Occupations exposed to whole body vibration for longer durations or in awkward postures appear to be associated with much higher risk of LBP |
| Nielsen, 2012[39]  (SR) | Back pain genetic phenotype heritability was 34% (95% CI, 30-39%) | Pain at other body sites | Heritability ranged from 0-68%. LBP with hospitalization has the greatest likelihood of heritability |
| Pincus, 2002[72]  (SR) | Psychological distress, depressive symptoms, depressive mood, and somatization associated with chronicity [NR] | NR | “Psychological factors (notably distress, depressive mood, and somatization) are implicated in the transition to chronic low back pain. The development and  testing of clinical interventions specifically targeting these factors is indicated.” |
| Ramond, 2011[73]  (SR) | Associated with transition from acute to chronic: Job satisfaction [NR]; compensation [NR]; coping strategies [NR]; fear avoidance behavior [NR]; patient expectations [NR] | NR | “Depression, psychological distress, passive coping strategies and fear-avoidance beliefs were sometimes found to be independently linked with the transition from acute to chronic low back pain.” |
| Ribeiro, 2012[74]  (SR) | > 2 hr spent in bent and twisted posture [RR = 2.21 (95% CI, 1.20-4.07)]; working in forward bent position > 60 min/day [OR = 8.7 (95% CI, 2.1-46)]. Insignificant ORs for the following: bending < 15 min, bending ­> 15 min, trunk flexion > 20^o^, bending to lift an item from the floor | NR | “We consider there is limited evidence for any association between quantity (ROM) or duration of trunk flexion and occupational LBP. We found no studies  and thus no evidence for frequency of trunk flexion as a risk factor for LBP.” |
| Roffey, 2010, 252-261[75]  (SR) | Occupational sitting: No studies demonstrated enough evidence to meet the Bradford Hill criteria for causality | NR | The authors found no compelling evidence of LBP causality from occupational sitting |
| Roffey, 2010, 639-651[76]  (SR) | Workplace manual handling or assisting patients: Only 7% of occupational postures were found to have statistically strong associations with LBP and 17% were found to be moderate. | NR | The evidence suggests that handling and assisting patients which result in awkward postures is statically unlikely to be the sole cause of LBP |
| Roffey, 2010, 89-99[77]  (SR) | Awkward occupational postures and any back pain: No studies demonstrated enough evidence to meet the Bradford Hill criteria for causality | NR | Awkward work postures were not likely to be the sole cause of LBP |
| Sitthipornvorakul, 2011[38]  (SR) | Physical activity: Heterogeneity in the studies population and methods prevented the authors from presenting statistical evidence | NR | No meaningful conclusion could be drawn on the association between physical activity and LBP |
| Taylor, 2014[78]  (SR) | Physical risk factors for first-ever reported LBP in community settings: Incidence 26% (95% CI 24% , 28% ) | NR | Pooled incidence of LBP in community and occupational settings is approximately 25% |
| Wai, 2010 pp. 554–566[79]  (SR) | Occupational lifting: a variety of risk estimates were deemed as being in conflict between studies. No measures of association were reported. | NR | “There was some moderate evidence for association for specific types of lifting and LBP, and some consistent evidence for association between lifting greater than 25 to greater than 35 kg and low back pain.” |
| Wai, 2010 pp. 628–638[80]  (SR) | Occupational carrying: several risk estimates were associated with decreased association with low back pain while others showed an increase in back pain. No measures of association were reported. | NR | “This systematic review found strong and consistent evidence against both an association and a temporal relationship  between occupational carrying and low back pain.” |
| Wai, 2010 pp. 76–88[81]  (SR) | Bending or twisting at various angles were associated with low back pain in some studies but not others. No measures of association were reported. | NR | “The evidence suggests that occupational  bending or twisting in general is unlikely to be independently causative of low back pain.” |
| Wertli, 2014, 2639 -2657[82]  (SR) | Catastrophizing  Heterogeneity of results was attributed to a lack of stratified cut offs and overall risk was not quantified | NR | There is some evidence that catastrophizing results in delayed recovery, but the relationship to LBP is not entirely clear. |
| Wertli, 2014, 816 – 83  6[83]  (SR) | Fear avoidance beliefs [OR range 1.05(95% CI, 1.02-1.09) to 2.63 (95%CI, 1.73-4.01)] | NR | High fear avoidance beliefs appear to be prognostic for poor outcomes in people with subacute LBP |
| Yassi, 2013[84]  (SR) | Nurses: working at an orthopedic clinic [RR = 55.2 (95% CI, 52.7–10.2]; greater than or equal to 1 patient transfer/shift (RR = 52.7 (95% CI, 51.6-4.5)]; working full-time [RR = 52.4 (95% CI 51.6-3.6)]. | NR | The authors conclude that there is evidence to suggest the nursing work duties may be related to the onset of nonspecific low back pain. |

^a^p = pooled measures of association from meta-analyses are denoted with a small case p (eg, pOR). Otherwise, reported measures of association are not pooled and are reported as results from individual studies reviewed.

^b^OR = odds ratio

^c^NR = not reported

^d^RR = relative risk

^e^LR = likelihood ratio
