# Supplementary material for: A scoping review of biopsychosocial risk factors and co-morbidities for common spinal disorders
Source: PLoS One. 2018 Jun 1;13(6):e0197987. doi: 10.1371/journal.pone.0197987 (PMC5983449; doi:10.1371/journal.pone.0197987)
Supplement: S3 Table — (DOCX) [file pone.0197987.s005.docx]

**Supplemental Table 3. Reported Risk Factors, Associations, and Comorbidities for Spinal Syndromes.**

| **Citation, year** | **Spinal Disorder** | **Risk Factor [Measure of Association]** | **Comorbidities Mentioned** | **Conclusion** |
| --- | --- | --- | --- | --- |
| Bovenzi, 1999[42]  (MA) | Sciatica | Whole body vibration from vehicles [pOR^a,b^ = 2.0 (95% CI, 1.3-2.9)] | NR^c^ | “…clear evidence for an increased risk for LBP disorders in occupations with exposure to WBV.” |
| Shiri, 2007[86]  (SR) | Lumbar radicular pain/sciatica | High BMI [NR]; long smoking history [NR]; high physical activity [NR]; high serum C-reactive protein level [NR] | NR | There is little evidence suggesting causal associations between these variables, mainly because those studied were covariates in the papers reviewed. The stage of the pathological process of these variables may be important in then being a determinant for sciatica modify. |
| Cook, 2014[85]  (SR) | Sciatica | Past history of LBP [overall OR = 3.1 (95% CI, 1.6-6.0), mild LBP [RR^d^ = 2.7 (95% CI, 1.7-4.2)], severe LBP [RR = 4.5 (95% CI, 2.7-7.6)], LBP > 3mths [female OR = 1.7 (95% CI, 1.2-1.6), male OR= 2.1 (95% CI, 1.2-3.8)]; drive for 2hrs > once per week [OR = 2.7 (95% CI, 1.2-6.4)]; bad self rated health [OR = 2.8 (95% CI, 1.2-7.1)]; manual labor [female OR = 1.5 (95% CI, 1.2-1.8), [male OR = 2.3 (95% CI, 1.7-3.2)]; overweight [female OR = 1.4 (95% CI, 1.2-1.6), HR^e^ = 7.1 (95% CI, 1.5-34.4), male OR = 1.3 (95% CI, 1.0-1.7)]; obese [female OR = 1.6 (95% CI, 1.3-1.9), male OR = 1.7 (5% CI, 1.1-2.4)]; smoking [previous OR=13.1 (95% CI, 2.7-65.1) and OR = 1.6 (95% CI, 1.1-2.4)], current [OR = 9.6, (95% CI, 1.7-53.0), current 1-15 daily cigarettes [OR = 1.9, (95% CI, 1.1-3.5)], current >15 daily cigarettes [OR = 2.5 (95% CI, 1.6-4.0)]; mental stress [some OR = 1.5 (95% CI, 1.0-2.4)], [much OR = 2.6 (95% CI, 1.5-4.5)]; physical exercise > once per week [RR = 1.3 (95% CI, 1.0-1.6)]; moderate twisting [OR = 1.7 (95% CI, 1.2-2.4)] much twisting [OR = 2.6 (95% CI, 1.7-4.1)]; kneeling/squatting > 1hr/day [OR = 2.6 (95% CI, 1.6-4.2); working with trunk flexed forward [OR = 2.1 (95% CI, 1.4-3.2)]; age 20-29 [RR = 2.7], 30-39 [RR = 8.6], 40-49 [RR = 12.1], 50-59 [RR = 7.3], > 60 [RR = 4.3] |  | All studies that investigated risk factors of occupations identified smoking and manual labor as risk factors. Non-modifiable risk factors are age and history of LBP. Modifiable risk factors for sciatica were obesity and smoking. |
| Carroll, 2008[87] | Whiplash | Passive coping predicted slower recovery [OR=0.45 (95% CI, 0.36-0.56)], especially in the presence of concurrent depression [OR = 0.25 (95% CI, 0.17-0.39)]; depression at 6 weeks predicted slower recovery [HRR^f^ = 0.68 (95% CI, 0.62-0.76)]; active coping did not predict time to recovery [OR = 1.08 (95% CI, 0.87-1.33)]; fitness training attended before 70 days of injury [HRR = .68 (95% CI .54-.86); outpatient rehabilitation attended before 120 days of injury [HRR =.50 (95% CI .33-.77)]; Fastest recovery times in those with 1-2 visits to general practitioners in the first month post-injury GP 1-2 days [HRR = 1.00], GP > 2 visits [HRR = 0.73 (95% CI, .61-.87)], DC > 6 visits [HRR = 0.61 (95% CI, .46-.81)], GP and specialist [HRR = 0.69 (95% CI, .55-.87)], Gen Med [HRR = 0.78 (95% CI, .64-.95)]. | Helplessness, depression, fear of movement,  catastrophizing, anxiety | Increased initial symptom severity is prognostic of poorer outcome. Collision factors did not seem to be associated with recovery. Passive coping (assessed at 6 weeks) was a strong and independent predictor of slowed recovery, especially in the presence of depression. |
| Holm, 2008[88] | Whiplash | Sex [IRR^g^=1.6 (95% CI 1.1-2.3), RR = 1.20 (95% CI, 1.16-1.25)];  age 18-34 yr vs 55+ [RR = 1.19 (95% CI, 1.11-1.28)]; age 35-44 yr vs 55+[ RR = 1.14 (95% CI, 1.06-1.23)]; age 45-54 yr vs 55+ [RR = 1.10 (95% CI, 1.02-1.18)]; during the last 6 months of tort insurance for age vs age 55+: 18-23 yr [IRR = 4.6 (95% CI NR)], age 24-29 [IRR = 3.3 (95% CI NR)], age 30-39 [IRR = 2.4 (95% CI NR)], age 40-49 [IRR = 1.9 (95% CI NR)]; during the first 2 periods (6 months each) of no-fault for age vs age 55+: 18-23 yr [IRR = 3.5 (95% CI NR)], age 25-29 [IRR = 2.5 (95% CI NR)], age 30-39 [IRR = 2.0 (95% CI NR)], age 40-49 [IRR = 1.8 (95% CI NR)]; rear-impact vs side-impact [RR=1.82 (95% CI, 1.68-1.96)]; front impact vs side impact [RR=1.25 (95% CI, 1.15-1.36)]; front impact vs other impacts [RR=1.17, 95% CI 1.07-1.27)]; driver vs rear seat passenger [RR = 1.78 (95% CI, 1.60-1.97)]; front seat passenger vs rear seat passenger [RR = 1.40 (95% CI, 1.25-1.57)] | NR | The evidence of whiplash determinants is sparse, but personal, societal, and environmental factors are of importance. Risk factors for whiplash associated disorders are not well established. |
| Scholten-Peeters, 2003[89] | Whiplash | Prognostic for persisting symptoms: High initial pain intensity [NR].  Not prognostic for persisting symptoms: older age [NR]; female gender [NR]; high acute psychological response [NR]; angular deformity of the neck [NR]; rearend  collision [NR]; compensation [NR]. | NR | “The presence or absence of these factors can help care providers to predict the prognosis and to guide treatment.” |
| Walton, 2013[90] | Whiplash | Not recovered at 6 mo post-injury vs recovered: Disability associated with Neck Disability Index greater than 15 [pOR = 42.18 (95% CI, 7.37-241.3)]; high neck pain intensity [pOR = 5.61 (95% CI, 3.74-8.43)]; catastrophizing [pOR = 3.77 (95% CI, 1.33-10.74)]; report of disturbed sleep since the accident [pOR = 2.96 (95% CI, 0.97-9.04)]; presence of any neck pain at inception (versus no neck pain) [pOR = 2.87 (95% CI, 1.51-5.46)]; presence of headache at inception [pOR = 2.70 (95% CI, 2.16-3.39)]; pain associated with Neck Disability Index less than 15 [pOR = 2.65 (95% CI, 1.59-4.39)]; Grades 2 or 3 whiplash associated disorders (versus 0 or 1) at 24 mo [pOR = 2.67 (95% CI, 1.99-3.58)]; Grade 3 whiplash associated disorders (versus 2) at 6 mo [pOR = 2.66 (95% CI, 1.54-4.58)]; restricted cervical range of motion [pOR = 2.56 (95% CI, 0.85-7.72)]; grade 3 whiplash associated disorders (versus 2) at 12 mo [pOR = 2.41 (95% CI, 1.62-3.59)]; grades 2 or 3 whiplash associated disorders (versus 0 or 1) at 6 mo [pOR = 2.40 (95% CI, 1.41-4.10)]; presence of radicular symptoms at inception [pOR = 2.09 (95% CI, 0.88-4.95)]; less than postsecondary education [pOR = 2.00 (95% CI, 1.60-2.51)]; no seatbelt used [pOR = 1.97 (95% CI, 1.17-3.32)]; grades 2 or 3 whiplash associated disorders (versus 0 or 1) at 12 mo [pOR = 1.96 (95% CI, 1.41-2.74)]; low back pain [pOR = 1.83 (95% CI, 1.25-2.67)]; preaccident history of headache [pOR = 1.81 (95% CI, 0.76-4.34)]; female [pOR = 1.64 (95% CI, 1.27-2.12)]; grade 3 whiplash associated disorders (versus 2) at 24 mo [pOR=1.63 (95% CI, 0.41-6.51)]; preaccident history of neck pain [pOR=1.59 (95% CI, 1.03-2.46)]; depressive symptoms pOR = 1.47 (95% CI, 0.71-3.03)]; obese body mass index category 1.24 (95% CI, 0.71-2.19)]; unprepared for the collision [OR = 1.14 (0.84-1.55)]; severe collision [pOR = 1.12 (95% CI, 0.90-1.39)]; front passenger in vehicle [pOR = 1.09 (95% CI, 0.78-1.53); rear-end collision [pOR = 1.07 (95% CI, 0.74-1.56); older age (greater than 50-55 y) [OR=1.00 (95% CI, 0.97-1.04)]; driver of vehicle [OR = 0.94 (95% CI, 0.69-1.28)]; no head restraint used [OR = 0.91 (95% CI, 0.63-1.32)]; vehicle stationary when hit [OR = 0.87 (95% CI, 0.61-1.25)]; frontal collision [OR = 0.79 (95% CI, 0.53-1.17); side or “other” collision [OR = 0.66 (95% CI, 0.27-1.59)] | NR | “12 of 28 variables have demonstrated statistically significant ability to predict recovered or not-recovered  group membership, with ORs ranging from small (preaccident history of neck pain, 1.59) to large (high pain intensity, 5.61; NDI, 42.18 when disability is also the outcome).” |
| Williams, 2007[91] | Whiplash | Poor vs good outcomes: High initial neck pain intensity [NR];  high initial neck pain related disability [NR]; cold hyperalgesia [NR] | Preinjury neck pain, back pain, headache, widespread chronic pain, degeneration; radicular symptoms, cranial nerve or brainstem disturbance, dizziness, dysphagia, fatigue, high body mass index | “Evidence suggests that increased initial pain intensity, pain related disability, and cold hyperalgesia are associated with a poorer outcome after a whiplash injury.” |
| Williamson, 2008[92] | Whiplash | Poor vs good outcomes: lower self-efficacy [NR]; greater post-traumatic stress [NR] | Depression, distress, stress, anxiety, coping,  self-efficacy, fear-avoidance beliefs, personality, cognitive  function, history of prior psychological or emotional  problems, wellbeing | Strength of studies was low with largely inconclusive results. |
| Aldabe, 2012 (pp. 1769–76)[94]  (SR) | pelvic girdle pain. | Greater pelvic width [NR]; morphology of symphysis pubis [NR]; greater pubic symphysis width [NR]; sacroiliac joint laxity [NR]; altered flexion-relaxation phenomenon [NR]; increased spinal and pelvic rotational motion [NR] |  | The evidence for association between PPGP and altered motor control and kinematics of the pelvis is moderate. |
| Aldabe, 2012 (pp. 1777–87)[93] | pelvic girdle pain | 6 studies included in review. Four ranked high, 2 ranked low quality. Among high quality studies 3 found no association between PPGP and relaxin levels. | NR | Level of evidence for association between PPGP and relaxin levels found to be low. |

^a^p = pooled measures of association from meta-analyses are denoted with a small case p (eg, pOR). Otherwise, reported measures of association are not pooled and are reported as results from individual studies reviewed.

^b^OR = odds ratio

^c^NR = not reported

^d^RR = relative risk

^e^HR = hazard ratio

^f^HRR = hazard rate ratio

^g^IRR = incidence rate ratio
