# Supplementary material for: A scoping review of biopsychosocial risk factors and co-morbidities for common spinal disorders
Source: PLoS One. 2018 Jun 1;13(6):e0197987. doi: 10.1371/journal.pone.0197987 (PMC5983449; doi:10.1371/journal.pone.0197987)
Supplement: S4 Table — (DOCX) [file pone.0197987.s006.docx]

**Supplemental Table 4. Reported Risk Factors, Associations, and Comorbidities for Arthritic Spinal Disorders.**

| **Citation, year** | **Spinal Disorder** | **Risk Factor [Measure of Association]** | **Comorbidities Mentioned** | **Conclusion** |
| --- | --- | --- | --- | --- |
| Bovenzi, 1999[42]  (MA) | Lumbar disc disorders | Whole body vibration from occupational vehicles [pOR^a,b^ = 1.8 (95% CI, 1.1-1.3)]; | NR^c^ | “…clear evidence for an increased risk for LBP disorders in occupations with exposure to WBV.” |
| Dario, 2015[44]  (MA) | Lumbar disc degeneration | Same-sex twins: BMI [pOR = 1.2 (95% CI, 1.1-1.4)]; dizygotic twins: BMI [pOR = 1.2 (95% CI, 1.2-1.7)]; monozygotic twins: BMI [pOR = 0.9 (95% CI, 0.6-1.3)]. | NR | Only 1 study showed potential association between lumbar disc degeneration and BMI. Genetics and early environment are possible mechanisms underlying the relationship between obesity and low back pain. |
| Ganko, 2015[95]  (MA) | Symptomatic disc disease | Bacterial disc infection vs no disc infection [pOR = 6.1 (95% CI, 1.4-25.9)] | NR | Bacterial disc infection associated with higher odds of symptomatic disc disease |
| Huang, 2016[96]  (MA) | Lumbar disc herniation | Tobacco smoking [RR^d^ = 1.27 (95 % CI, 1.15-1.40)] | NR | Statistically significant association between lumbar disc herniation and tobacco smoking |
| Pabalan, 2016[97]  (MA) | Intervertebral disc degeneration | Vitamin D receptor FokI polymorphism [pOR = 0.96-1.04 (p = 0.73-0.95)]; non-Hispanic Caucasians subgroup [pOR = 0.77 (p = 0.01)]; male subgroup [pOR = 0.36–0.66 (p = 0.001–0.04)]; Hispanic Caucasians subgroup [pOR = 1.39-1.85 (p = 0.006–0.05)]; female subgroup [pOR = 1.72 (p = 0.05)]. Vitamin D receptor ApaI polymorphism [pOR = 0.69 (p = 0.04)]; Asian subgroup [pOR = 0.75–0.93 (p = 0.17-0.74)]. | NR | “This metaanalysis confirmed the protective role of the ApaI polymorphism, however, susceptibility and protective effects of the FokI polymorphism may be ethnic and gender specific.” |
| Raastad, 2015[98]  (MA) | Lumbar spine degeneration associated pain | Community-based samples: disc space narrowing [pOR = 1.47 (95% CI, 1.36-1.58)]; osteophytes [pOR = 1.20 (95% CI, 1.06-1.37)]; spondylolisthesis [pOR = 1.12 (95% CI, 1.03-1.23)]; spondylosis [pOR = 1.32 (95% CI, 1.14-1.53)]; end plate sclerosis [pOR = 1.24 (95% CI, 0.84-1.81)]; facet joint osteoarthritis [pOR = 1.07 (95% CI, 0.63-1.80)].  Occupation-based samples: disc space narrowing [pOR = 1.76 (95% CI, 1.34-2.33)]; osteophytes [pOR = 1.83 (95% CI, 0.88-3.79)]; spondylolisthesis [pOR = 2.21 (95% CI, 1.44-3.39)]; end plate sclerosis [pOR = 1.13 (95% CI, 0.78-1.65)]. | NR | As determined by findings on conventional radiographs, disc space narrowing and spondylolisthesis were associated with low back pain in both community and occupation-based samples, with spondylolisthesis significantly more prevalent in the occupations. Osteophytes and spondylosis were associated with low back pain in community-based studies but not in occupation-based studies. End plate sclerosis and facet joint osteoarthritis were not associated with low back pain in either sample type. |
| Shiri, 2015[27]  (MA) | Cervical disc degeneration  Lumbar disc degeneration | Fighter pilot vs helicopter or transport pilot [pOR = 1.26 (95% CI, 0.81-1.96)] vs non-flying personnel [pOR = 1.14 (95% CI, 0.61-2.16)]; Fighter pilot vs helicopter or transport pilot [pOR = 0.87 (95% CI, 0.67-1.13)] vs non-flying personnel [pOR = 1.05 (95% CI, 0.49-2.26)] | NR | No difference in cervical or lumbar degeneration based on aircraft platform and no difference between flying personnel and non-flying personnel |
| Faraj, 2016[99]  (SR) | Degenerative scoliosis curve progression | Increasing intervertebral  disk degeneration [NR]; lateral vertebral translation > 6 mm [NR]; intercrest line through L5 [NR] | NR | “This review shows strong evidence that increased intervertebral  disk degeneration, an intercrest line through L5,  and lateral vertebral translation C6 mm are associated with  curve progression.” |
| Hogg-Johnson, 2008[29]  (SR) | Cervical disc protrusion | Male vs. female [IRR^e^ = 1.41 (95% CI, 0.84–2.39)] | NR | Male sex associated with higher incidence of cervical radicular symptoms in presence of cervical disc protrusion |
| Wong, 2014[100]  (SR) | Cervical disc herniation | Workers’ compensation claim may be related to poorer prognosis [NR] | NR | Most patients with symptomatic cervical  spine disc herniation with radiculopathy recover. |

^a^p = pooled measures of association from meta-analyses are denoted with a small case p (eg, pOR). Otherwise, reported measures of association are not pooled and are reported as results from individual studies reviewed.

^b^OR = odds ratio

^c^NR = not reported

^d^RR = relative risk

^e^IRR = incidence rate ratio
