# Supplementary material for: A scoping review of biopsychosocial risk factors and co-morbidities for common spinal disorders
Source: PLoS One. 2018 Jun 1;13(6):e0197987. doi: 10.1371/journal.pone.0197987 (PMC5983449; doi:10.1371/journal.pone.0197987)
Supplement: S5 Table — (DOCX) [file pone.0197987.s007.docx]

**Supplemental Table 5. Reported Risk Factors, Associations, and Comorbidities for Traumatic Spinal Disorders.**

| **Citation, year** | **Spinal Disorder** | **Risk Factor [Measure of Association]** | **Comorbidities Mentioned** | **Conclusion** |
| --- | --- | --- | --- | --- |
| Chamberlain, 2015[113]  (MA) | Spinal cord injury | For mortality associated with SCI: Increasing age [pHR^a,b^ = 1.06 (95% CI, 1.05–1.07)]; male sex [pHR = 1.29 (95% CI, 1.21–1.36)]; higher level of lesion [NR^c^]; completeness of lesion [NR] | NR | Reduction of inequities and discrepancies, both within the SCI population as well as compared to the general population, could be achieved through focused  management of SCI-specific health issues |
| Craig, 2009[101]  (SR) | Spinal cord injury | NR | Depression, depressive symptoms, depressive mood, major depressive disorder, anxiety, post-traumatic stress disorder, reduced quality of life, high levels of pain | Spinal cord injuries are associated with a number of psychiatric comorbidities. It is unknown how many people with SCI have psychological disorders because of their medical condition or if it is they are comorbid variable. |
| Dijkers, 2009[102]  (SR) | Traumatic spinal cord injury | NR | Chronic pain | Chronic pain (location not specified) is a common comorbidity following traumatic spinal cord injury and may vary by sex, level of spinal cord injury, and completeness of injury |
| Gilbert, 2014[103]  (SR) | Spinal cord injury | Cardiovascular disease [NR] | NR | Adults with spinal cord injury have lower total cholesterol, lower high-density lipoprotein cholesterol, and higher total cholesterol/high-density lipoprotein cholesterol ratios compared to able-bodied individuals |
| Jazayeri, 2015[104]  (SR) | Traumatic spinal cord injury | motor vehicle  accidents, falls, violence, and sports injuries [NR] | NR | Incidence of SCI ranged from 3.6 per million in Canada to 195.4 per million in Ireland Most SCI cases were due to trauma, including traffic accidents and falls. |
| Kraft, 2015[105]  (SR) | Spinal cord injury | NR | Depression, depressive symptoms | Depressive individuals reported learned helplessness, lowered self-efficacy and self-control, distorted representations of SCI-related disability, heightened stress, fewer vocational interests and skills, reduced sense of hope, pain catastrophizing, alcohol and drug misuse, reduced problem-solving abilities, less enjoyment from experiences or hobbies, feelings of social isolation, greater dissatisfaction with the quantity and quality of available social and community supports |
| Lidal, 2007[106]  (SR) | Spinal cord injury | Risks for returning to work following spinal cord injury: injury at older age [NR], severe injury [NR], low functional independence [NR] | NR | There are high  unemployment rates in individuals with spinal cord injury. |
| Ning, 2012[107]  (SR) | Spinal cord injury | Male sex [NR], motor vehicle accidents [NR], falls [NR] | NR | Because of limitations in how papers are reported it was difficult to establish accurate epidemiological data for spinal cord injury in Asia. |
| Parent, 2011[108]  (SR) | Spinal cord injury | Motor vehicle accidents more common cause for children [NR] and sports injuries in adolescents [NR]. All-terrain vehicle accidents more common cause of injury in children than adults [NR] | Scoliosis | Pediatric patients with spinal cord injury have different characteristics than adults with spinal cord injury |
| van den Berg, 2010 (pp 1517–1528)[109]  (SR) | Spinal cord injury | Risk of mortality higher for older age at lesion onset [NR]; higher neurological level [NR]; completeness of injury [NR]; non-traumatic spinal cord injury [NR]. | NR | General mortality rates of individuals with spinal cord injury exceed those of the age-matched non-disabled population, mainly due to secondary complications. |
| van den Berg, 2010 (pp 184–192 )[110]  (SR) | Spinal cord injury | Traumatic: motor vehicle collisions [NR], falls [NR], violence [NR], sports activities [NR], female vs male [IRR^d^ = 0.33 (95% CI, 0.26-0.41]; age 20-29 yr [IRR = 6.07 (95% CI, 3.72-9.91]; age > 70 yr [IRR = 9.0 (95% CI, 4.40-14.92].  Non-traumatic: female vs male [IRR = 0.39 (95% CI, 0.29-0.53]; increasing age of 75-84 yr [IRR = 5.22 (95% CI, 2.90-9.39.] | NR | An increase in the incidence of non-traumatic spinal cord injury is anticipated with the increasing age of the population |
| Van Gorp, 2015[114]  (SR) | Spinal cord injury | Association of pain of spinal cord injury: time since injury [NR]; depression [NR] | Depression | Variance in SCI pain prevalence is mostly due to differences in the strictness of the pain definition and whether or not the study was a pain study. |
| van Middendorp, 2010[111]  (SR) | Cervical spine fracture or dislocation | Risk of mortality higher for those with spinal cord injury [NR]; increasing age[NR]; pre-existing comorbidities [NR]; ankylosing spondylitis [NR]; higher injury score [NR] | Cardiovascular diseases, ankylosing spondylitis, osteoarthritis, metabolic disorders, pulmonary disease, gastrointestinal disease, renal pathology, neoplasia, cerebral pathology | Reporting methods in the studies reviewed were inadequate for drawing valid conclusions |
| Wilson, 2012[115]  (SR) | Spinal cord injury | Better neurologic outcomes associated with incomplete injury and zone of partial preservation. Severe patterns  of neurological injury, more cephalad neurological level of injury, increasing age, associated with higher mortality rates. | NR | A constellation of clinical  features relating to neurological examination, demography, and injury etiology help to more  accurately define profiles for recovery and survival. |
| Xing, 2013[112]  (SR) | Cervical spine fracture or dislocation | Risk of mortality higher for those with spinal cord injury [NR]; pre-existing comorbidities [NR]; older age [NR]; closed head injury [NR]; higher injury severity score [NR]; cardiopulmonary disease [NR] | Peripheral neuropathy, cardiopulmonary disease, low bone mass, reduced lean muscle mass | Age, pre-existing comorbidities, and presence of spinal cord injury considered “strong evidence prognostic predictors” for mortality associated with cervical spine injury. |

^a^p = pooled measures of association from meta-analyses are denoted with a small case p (eg, pOR). Otherwise, reported measures of association are not pooled and are reported as results from individual studies reviewed.

^b^HR = hazard ratio

^c^NR = not reported

^d^IRR = incidence rate ratio
