# Supplementary material for: A scoping review of biopsychosocial risk factors and co-morbidities for common spinal disorders
Source: PLoS One. 2018 Jun 1;13(6):e0197987. doi: 10.1371/journal.pone.0197987 (PMC5983449; doi:10.1371/journal.pone.0197987)
Supplement: S6 Table — (DOCX) [file pone.0197987.s008.docx]

**Supplemental Table 6. Reported Risk Factors and Comorbidities for Spinal Tuberculosis.**

| **Citation, year** | **Risk Factor [Measure of Association]** | **Comorbidities Mentioned** | **Conclusion** |
| --- | --- | --- | --- |
| Alavi, 2010[116]  (case-control) | Age > 35 yr [OR^a^ = 4.7 (95% CI, 2.3-9.7)]; history of imprisonment [OR = 2.9 (95% CI, 1.3-6.6)]; chronic renal failure [OR = 2.7 (95% CI, 1.1-7.3)]; male [OR = 1.9 (95% CI, 1.1-3.4)]; history of previous TB [OR = 1.9 (95% CI, 1.5-3.9)]; diabetes mellitus [OR = 1.4 (95% CI, 0.5-4.0)]; history of corticosteroid [OR = 1.6 (95% CI, 0.6-3.9)]; intravenous drug user [OR = 1.4 (95% CI, 0.6-3.1)]; HIV co-infection [OR = 1.3 (95% CI, 0.6-2.9)] | Chronic renal failure, diabetes mellitus, HIV | Older age, male gender, chronic peritoneal dialysis, imprisonment  and previous TB are associated with higher risk of spinal tuberculosis. |
| Gao, 2014[117]  (case-control) | Monocyte chemotactic protein-1 (MCP-1) -2518 GG genotype [OR = 2.306 (95% CI, 1.273-4.178)] | NR | “The MCP-1 -2518 GG genotype and presence of the G allele may be associated  with susceptibility to spinal tuberculosis in the Chinese Han population.” |
| Guo, 2014[118]  (case-control) | Monocyte chemoattractant protein-1 (MCP-1) -362GC genotype [OR = 1.376 (95% CI, 1.109-1.706)] | NR | The MCP-1-362G/C genetic variant was associated with spinal TB in the Han Chinese population |
| Mecabih, 2016[119]  (case-control) | Monocyte chemoattractant protein-1 (MCP-1) – 2518GG (rs1024611) [OR = 3.20 (95% CI, 1.30–8.41); MCP1-362CC (rs2857656) [OR = 2.53 (95% CI, 1.10-5.96)]; MCP1 deldel554-567 (rs3917887) [OR = 2.59 (95% CI, 1.10-6.34)] | NR | Findings confirm and replicate data from China and provide trans-ethnic evidence of the association. |
| Rajasekaran, 2007[120]  (cohort) | Clinical findings correlating to progression severe vertebral collapse: age < 7 yr at time of diagnosis [NR]; involvement at thoracolumbar level [NR]; loss of > 2 vertebral bodies [NR]; presence of at-risk signs on radiography [NR] | NR | Findings help as prognostic factors for surgery candidacy. |
| Rajasekaran, 2006[122]  (cohort) | Children younger than 10 years differed from those 11 years or older by having significantly more severe  disease and more number of morphological changes with growth in both the fusion mass and the adjacent segments. | NR | “In many parts of the world where childhood spinal tuberculosis is endemic, children are unfortunately routinely discharged from care 2 to 3 years after complete cure of the disease is achieved. This leads to a high risk that many children may deteriorate in deformity  unobserved. The results of our study clearly point out that every child with spinal tuberculosis must be routinely observed until the entire growth potential is completed.” |
| Rajasekaran, 2001[123]  (cohort) | A radiographic instability score >2 of dislocation of the facets, posterior retropulsion of the diseased fragments, lateral translation of the vertebrae in the anteroposterior view and toppling of the superior vertebra was a predictor of patients with an increase of more than 30° in deformity and a final deformity of over 60° at 15 year follow up (p<.0001) | NR | Signs of radiological instability appear early in spinal TB and can be reliably used to identify  children whose spine is at risk for late progressive collapse, thus surgery is advised in these cases. |
| Rajasekaran, 1998[121]  (cohort) | Children < 10 yr old had worse kyphosis, an average involvement of 3.1 vertebral bodies and average vertebral loss of 2.2 in comparison to > 17 yr olds (1.9 levels involved, .87 lost, p < .01) | NR | Children < 10 yr have more severe involvement with increased tendency toward hyperkyphosis |

^a^OR = odds ratio

^b^NR = not reported
