# Supplementary material for: A scoping review of biopsychosocial risk factors and co-morbidities for common spinal disorders
Source: PLoS One. 2018 Jun 1;13(6):e0197987. doi: 10.1371/journal.pone.0197987 (PMC5983449; doi:10.1371/journal.pone.0197987)
Supplement: S7 Table — (DOCX) [file pone.0197987.s009.docx]

**Supplemental Table 7. Reported Risk Factors, Associations, and Comorbidities for Metabolic Spinal Disorders.**

| **Citation, year** | **Spinal Disorder** | **Risk Factor [Measure of Association]** | **Comorbidities Mentioned** | **Conclusion** |
| --- | --- | --- | --- | --- |
| Avenell, 2014[124]  (MA) | Vertebral fracture | For all fractures, vitamin D + calcium [pR/R^a,b^ = 0.95 (95% CI, 0.90-0.99)]; for new vertebral fracture or deformity [Pr/R = 0.89 (95% CI, 0.74-1.09)] | NR | Vitamin D + calcium reduces the risk of any type of fracture but not specifically new vertebral fracture or deformity. “only moderate quality evidence of an absence of a statistically significant preventive effect on clinical vertebral fractures” |
| Biver, 2011[125]  (MA) | Vertebral fracture | Leptin [pOR^d^ = 0.642 (95% CI, 0.429-0.960)];  Adiponectin in men [paOR = 1.13 (95% CI, 1.08-1.23)];  Adiponectin levels greater than 10 mg/ml in men vs lower levels [paOR = 2.98 (95% CI, 1.08-8.26)]. | Bone mineral density | “The influence of plasma leptin, adiponectin, and ghrelin levels on BMD values would be very weak and mediated or confounded by the specific body composition parameters.” “High levels of leptin were reported to be predictive of low risk of fractures, whereas high levels of adiponectin may be predictive of high risk of vertebral fractures in men only.” |
| Bolanos, 2010[126]  (MA) | Vertebral Fracture | Hormone therapy and isoflavones:  [pOR =1.56 (95% CI, 0.39-6.19)] | NR | No significant difference between hormone therapy or isoflavones in reduction of vertebral fractures |
| Bolland, 2015[127]  (MA) | Vertebral fracture | Calcium supplements [pRR^e^ = 0.86 (95% CI, 0.74-1.00)] | Frail | “Overall, there is little evidence currently to suggest an association between calcium intake and fracture risk or that increasing calcium intake through dietary sources will alter risk.” “In summary, our analyses indicate that dietary calcium intake is not associated with risk of fracture, and there is no evidence currently that increasing dietary calcium intake prevents fractures.” |
| Brennan, 2011[146]  (SR) | Bone mineral density | Education was associated with higher lumbar BMD [OR = 0.76 (95% CI, 0.65-0.90)] | NR | “Limited good quality evidence exists for the role that education level may play in BMD levels.” |
| Cockayne, 2006[128]  (MA) | Vertebral Fracture | Supplementation with phytonadione and menaquinone-4 (Vitamin K): [pOR =0.40 (95% CI, 0.25-0.65)] | NR | Supplementation with phytonadione and menaquinone-4 reduces bone loss and in Japanese patients taking menaquinone-4, reduction in fractures |
| D’Sylva, 2015[129]  (MA) | Vertebral Fracture | Hyperprolactinemia  women [pOR=0.29 (95% CI, 0.10-0.78)]; men [pOR=0.18 (95% CI, 0.03-0.94)]; combined [pOR=0.25 (95% CI, 0.11-0.59)] | NR | Increased fractures in patients with untreated hyperprolactinemia |
| Jackson, 2007[130]  (MA) | Vertebral Fracture | Vitamin D3 preventing vertebral fractures:  [pRR = 1.22 (95% CI, 0.64-2.31)] | NR | Did not clearly demonstrate reduction of vertebral fractures |
| Kemmler, 2013[131]  (MA) | Vertebral Fracture | Exercise: [pRR =0.56 (95% CI, 0.30-1.04)] | NR | “…although there is a strong body of evidence, we were still unable to provide the definite proof that exercise positively impacts overall fractures or vertebral fractures.” |
| Lock, 2006[132]  (MA) | Vertebral Fracture | Exercise: [pR/R = 0.52 (95% CI, 0.17-1.60)] | NR | Exercise showed a non-significant reduction of spinal fractures |
| Ngamruengphong, 2011[133]  (MA) | Vertebral Fracture | Use of proton pump inhibitor vs not: [pOR=1.50 (95% CI, 1.32-1.72)] | NR | Potential association between PPIs and risk of vertebral fractures |
| O’Donnell, 2008[134]  (MA) | Vertebral Fracture | Vitamin D (calcitriol and alfacalcidol) [pOR=0.89 (95% CI, 0.57-1.39)]; (alfacalcidol only):  [pOR=0.50 (95% CI, 0.25-0.98)] | NR | Combined results of 13 trials showed vertebral fractures were not significantly reduced. However, 5 trials showed significant reduction in vertebral fractures with the use of alfacaldidol |
| Rabenda, 2013[135]  (MA) | Vertebral Fracture | SSRIs [pRR =1.22 (95% CI, 1.05-1.42)]; anti-depressants [pRR =1.38 (95% CI, 1.19-1.61)] | NR | Use of SSRIs and anti-depressants were associated with an increase in spine fractures |
| Reid, 2014[136]  (MA) | Lumbar spine bone mineral density | Vitamin D [pWMD^f^ = 0.0 (95% CI, –0.2-0.3)] | NR | “no significant effect of vitamin D on bone  mineral density in either the spine” |
| Richards, 2009[137]  (MA) | Lumbar spine bone mineral density and vertebral fracture | Single nucleotide  polymorphisms from the LRP5, SOST, SPP1, and TNFRSF11A loci were significantly associated with fracture risk [OR ranged from 1.13-1.43 per allele] | NR | “the 9 loci identified, which influence BMD and possibly fracture risk, may have potential clinical utility if medicines can be safely used to influence their function.” |
| Ruyssen-Witrand, 2007[138]  (MA) | Vertebral fracture | Greater vertebral depth from T8 to L1 [pOR = 1.38 (95% CI, 1.09-1.75)]; larger vertebral area [pOR = 1.46, 95% CI, 1.03-2.06)]; greater depth T4-L5 [pOR = 1.31 (95% CI, 1.01-1.71)]; greater area T4-L5 [pOR = 1.46 (95% CI, 1.03-2.06)]; volume controlled for factors including age, and fracture risk:50−59 yr L1-L3 mean [pOR = 2.13 (95% CI, 0.75-3.45)]; 60–69 yr L1-L3 mean [pOR = 0.64 (95% CI, 0.43-1.03)]; 70–79 yr L1-L3 mean [pOR = 1.64 (95% CI, 0.29-9.09)]. | NR | Small vertebral body dimensions were associated with increased risk for osteoporotic vertebral fracture |
| Shah, 2015[139]  (MA) | Vertebral fracture | Type 1 diabetes [pRR = 2.88 (95% CI, 1.71-4.82)] | NR | “type 1 DM might be associated with increased risk for any fractures. The risk for fracture is higher in both men and women with type 1 DM.” |
| Tai, 2015[140]  (MA) | Lumbar spine bone mineral density | Calcium intake from  dietary sources: 1 yr [pWMD = 0.6 (95% CI, −0.1-1.3)]  2 yr [pWMD = 0.7 (95% CI, 0.3-1.2)]. Supplements: 1 yr [pWMD = 1.2 (95% CI, 0.8-1.7)]; 2 yr [pWMD = 1.1 (0.7 to 1.6)]; > 2.5 yr [pWMD = 1.0 (95% CI, 0.3-1.6)] | NR | Dietary calcium and calcium supplements have little effect on BMD for those 50+ years of age |
| Veronese, 2015[141]  (MA) | Vertebral fracture | Vitamin K antagonist medications: Cross sectional studies [pR/R = 1.75 (95% CI, 0.78-3.92)]; Longitudinal studies [pRR = 1.20 (95% CI, 0.81-1.76)] | NR | People treated with Vitamin K antagonists did not have an  increased risk of  new vertebral fractures |
| Vestergaard, 2003[144]  (MA) | Vertebral fracture | Current smoker [pRR = 1.76 (95% CI, 1.10-2.82)] | NR | Fracture risk was significantly increased in current smokers but not former smokers |
| Vestergaard, 2007[142]  (MA) | Vertebral fracture | Type 1 or Type 2 diabetes [pRRk = 0.93 (95% CI, 0.63-1.37)]; BMI [R^2^ = 0.34 (±0.10)] |  | Type 1 or Type 2 diabetes were not associated with an increase or decrease in vertebral fracture risk.  Meta-regression showed that body mass index was a determinant for BMD in the spine for those with Type 2 but not Type 1 diabetes mellitus |
| Vestergaard, 2008[143]  (MA) | Vertebral Fracture | Fluoride [pOR = 0.8 (95%CI, 0.5 to 1.3)]; Low daily dose Fluoride </=20mg fluoride equivalents [pOR = 0.28 (95%CI, 0.09- 0.87)] | NR | Overall, no significant reduction in vertebral fracture risk; however, in subgroup analyses of low dose fluoride there was a significant reduction in vertebral fractures |
| Winzenberg, 2006[145]  (MA) | Lumbar spine bone mineral density | Calcium supplementation [pooled standardized mean difference = 0.08 (95% CI, -0.04-0.20)] | NR  (only healthy children included) | Calcium supplementation has no effect on spine BMD in healthy children |

^a^p = pooled measures of association from meta-analyses are denoted with a small case p (eg, pOR). Otherwise, reported measures of association are not pooled and are reported as results from individual studies reviewed.

^b^R/R = risk ratio

^c^NR = not reported

^d^OR = odds ratio

^e^RR = relative risk

^f^WMD = weighted mean diff
