# Supplementary material for: A scoping review of biopsychosocial risk factors and co-morbidities for common spinal disorders
Source: PLoS One. 2018 Jun 1;13(6):e0197987. doi: 10.1371/journal.pone.0197987 (PMC5983449; doi:10.1371/journal.pone.0197987)
Supplement: S8 Table — (DOCX) [file pone.0197987.s010.docx]

**Supplemental Table 8. Reported Risk Factors, Associations, and Comorbidities for Congenital/Developmental Spinal Disorders.**

| **Citation, year** | **Spinal Disorder** | **Risk Factor [Measure of Association]** | **Comorbidities Mentioned** | **Conclusion** |
| --- | --- | --- | --- | --- |
| Dean, 2014[148]  (MA) | Neural tube defects | Folic acid supplementation and risk of neural tube defects:  RCTs only [pRR^a,b^ = 0.31 (95% CI, 0.14-0.66)]; RCT plus observational studies [pRR = 0.43 (95% CI, 0.13-1.40)] | NR | Folic acid supplementation has a strong protective effect against neural tube defects when studies are RCTS, the effect is not significant when observational studies are included. |
| Ford, 2014[149]  (MA) | Neural tube defects | First-trimester efavirenz exposure (antiretroviral medication used to treat and prevent HIV/AIDS). Overall congenital anomaly [pR R = 0.78 (95% CI, 0.56–1.08)]; “incidence of neural tube defects was low, 0.05% (95% CI <0.01–0.28), and similar to incidence in the general population.” | NR | No increased risk for neural tube defect by taking efavirenz during first trimester |
| Hwang, 2003[150]  (MA) | Neural tube defects | Prenatal exposure of chlorine byproducts in drinking water on neural tube defect [pOR^d^ = 1.49 (95% CI, 1.08, 2.05)] | NR | Prenatal exposure to chlorine increases risk of neural tube defects.  There is increased risk of neural tube defects with prenatal exposure to chlorine (through drinking water) |
| Jentink, 2010[151]  (MA) | Spina bifida | Carbamazepine (medication to treat epilepsy and neuropathic pain) effect on neural tube defect [pOR = 2.6 (95% CI, 1.2, 5.3)] | NR | “Spina bifida was the only specific major congenital malformation significantly associated with exposure to carbamazepine  monotherapy… compared with no antiepileptic drug” |
| Jentink, 2010[152]  (MA) | Spina bifida | Valproic acid effect on neural tube defect [pOR = 12.7 (95% CI, 7.7-20.7)] | NR | Valproic acid during first trimester increases the risk of spina bifida |
| Luteijn, 2014[153]  (MA) | Neural tube defects | First trimester maternal influenza exposure. All neural tube defects [pOR = 3.33 (95% CI, 2.05-5.40)] | NR | “One of the most striking results of the meta-analysis is the association  between first trimester influenza exposure and neural tube defects.” |
| Luteijn, 2014[153]  (MA) | Spina bifida | First trimester maternal influenza exposure. Spina bifida [pOR = 2.20 (95% CI, 1.48-3.28)] | NR | “One of the most striking results of the meta-analysis is the association between first trimester influenza exposure and neural tube defects.” |
| Stothard, 2009[154]  (MA) | Spina bifida | Maternal obesity [pOR = 2.24 (95% CI, 1.86-2.69)] | NR | Maternal obesity increases risk for spina bifida |
| Stothard, 2009[154]  (MA) | Neural tube defects | maternal obesity [pOR = 1.87 (95% CI, 1.62-2.15)]; maternal overweight [pOR = 1.20 (95% CI, 1.04-1.38)] | NR | Maternal obesity and material overweight, increases risk for neural tube defects |
| Tanoshima, 2015[155]  (MA) | Neural tube defects | Valproic acid [pRR = 7.44 (95% CI, 4.71-11.75)] | NR | Exposure to valproic acid increases the risk of neural tube defects |
| Chen, 2014[156]  (MA) | Adolescent idiopathic scoliosis | Polymorphism rs9340799 of the estrogen receptor alpha gene [pOR = 1.09 (95% CI, 0.96-1.23)] | NR | “The rs9340799 polymorphism does not appear to be a likely susceptibility variant for AIS predisposition.” |
| Noshchenko, 2015[157]  (MA) | Adolescent idiopathic scoliosis progressive severe deformity | High initial Cobb angle [pOR = 7.6 (95% CI, 4.2-13.6)]; thoracic curve [pOR = 2.3 (95% CI, 1.2-4.6)]; osteopenia [pOR = 2.6 (95% CI, 1.4-5.6)]; age < 13 yr at time of diagnosis [pOR = 2.7 (95% CI, 1.9-3.9)]; pre-menarche at diagnosis [pOR = 4.0 (95% CI, 2.0-7.9)]. | NR | “This review did not reveal any methods for the prediction of progression in AIS that could be recommended for clinical use as diagnostic criteria.” |
| Yang, 2015[158]  (MA) | Adolescent idiopathic scoliosis | rs4753426 polymorphism [pOR = 1.12 (95% CI, 1.03-1.21)]; C allele significant in Asians but not in whites | NR | “This meta-analysis found an overall significant association  between rs4753426 and AIS, especially in the Asian population.” |

^a^p = pooled measures of association from meta-analyses are denoted with a small case p (eg, pOR). Otherwise, reported measures of association are not pooled and are reported as results from individual studies reviewed.

^b^RR = relative risk

^c^NR = not reported

^d^OR = odds ratio
